# Ligand-independent activation of platelet-derived growth factor receptor β promotes vitreous-induced contraction of retinal pigment epithelial cells

**DOI:** 10.1186/s12886-023-03089-8

**Published:** 2023-08-03

**Authors:** Yajian Duan, Wenyi Wu, Jing Cui, Joanne Aiko Matsubara, Andrius Kazlauskas, Gaoen Ma, Xiaorong Li, Hetian Lei

**Affiliations:** 1 Tianjin Key Laboratory of Retinal Functions and Diseases, Tianjin Branch of National Clinical Research Center for Ocular Disease, Eye Institute and School of Optometry, Tianjin Medical University Eye Hospital, Tianjin, China; 2 Shanxi Bethune Hospital, Shanxi Academy of Medical Sciences, Tongji Shanxi Hospital, Third Hospital of Shanxi Medical University, Taiyuan, China; 3 Tongji Hospital, Tongji Medical College, Huazhong University of Science and Technology, Wuhan, 430030 China; 4 Department of Ophthalmology, Hunan Key Laboratory of Ophthalmology, National Clinical Research Center for Geriatric Disorders, Xiangya Hospital of Central South, Changsha, China; 5 Department of Ophthalmology and Visual Sciences, The University of British Columbia, Vancouver, Canada; 6 Department of Ophthalmology, University of Illinois at Chicago, Chicago, USA; 7 Department of Ophthalmology, the Third Affiliated Hospital of Xinxiang Medical University, Xinxiang, 453000 China

**Keywords:** Vitreous, Indirect activation, PDGFRβ, Akt, Retinal pigment epithelial cells, Proliferation, Epithelial-mesenchymal transition, Migration, Contraction

## Abstract

**Background:**

Epiretinal membranes in patients with proliferative vitreoretinopathy (PVR) consist of extracellular matrix and a number of cell types including retinal pigment epithelial (RPE) cells and fibroblasts, whose contraction causes retinal detachment. In RPE cells depletion of platelet-derived growth factor (PDGF) receptor (PDGFR)β suppresses vitreous-induced Akt activation, whereas in fibroblasts Akt activation through indirect activation of PDGFRα by growth factors outside the PDGF family (non-PDGFs) plays an essential role in experimental PVR. Whether non-PDGFs in the vitreous, however, were also able to activate PDGFRβ in RPE cells remained elusive.

**Methods:**

The CRISPR/Cas9 technology was utilized to edit a genomic *PDGFRB* locus in RPE cells derived from an epiretinal membrane (RPEM) from a patient with PVR, and a retroviral vector was used to express a truncated PDGFRβ short of a PDGF-binding domain in the RPEM cells lacking PDGFRβ. Western blot was employed to analyze expression of PDGFRβ and α-smooth muscle actin, and signaling events (p-PDGFRβ and p-Akt). Cellular assays (proliferation, migration and contraction) were also applied in this study.

**Results:**

Expression of a truncated PDGFRβ lacking a PDGF-binding domain in the RPEM cells whose *PDGFRB* gene has been silent using the CRISPR/Cas9 technology restores vitreous-induced Akt activation as well as cell proliferation, epithelial-mesenchymal transition, migration and contraction. In addition, we show that scavenging reactive oxygen species (ROS) with N-acetyl-cysteine and inhibiting Src family kinases (SFKs) with their specific inhibitor SU6656 blunt the vitreous-induced activation of the truncated PDGFRβ and Akt as well as the cellular events related to the PVR pathogenesis. These discoveries suggest that in RPE cells PDGFRβ can be activated indirectly by non-PDGFs in the vitreous via an intracellular pathway of ROS/SFKs to facilitate the development of PVR, thereby providing novel opportunities for PVR therapeutics.

**Conclusion:**

The data shown here will improve our understanding of the mechanism by which PDGFRβ can be activated by non-PDGFs in the vitreous via an intracellular route of ROS/SFKs and provide a conceptual foundation for preventing PVR by inhibiting PDGFRβ transactivation (ligand-independent activation).

**Supplementary Information:**

The online version contains supplementary material available at 10.1186/s12886-023-03089-8.

## Background

Platelet-derived growth factor (PDGF) receptors (PDGFRs) were in 1994 firstly shown to be expressed in cells within epiretinal membranes (ERMs) from patients with proliferative vitreoretinopathy (PVR) [1]. PVR is a fibrotic eye disease, which develops at a rate of 5–10% after surgery correction of a retinal detachment [2–4] and occurs at a rate of 40–60% after open ocular trauma [5, 6]. In the PVR pathogenesis retinal cells including retinal pigment epithelial (RPE) cells lodged in the vitreous after retinal repairing surgery or mechanistic retinal damage form epi- or sub-retinal membranes (ERMs) after their proliferation, epithelial-mesenchymal transition (EMT), migration and secretion of extracellular matrix [3, 7]. The tracking force of the ERMs causes retinal detachment [8]. At present there is no approved medicine for this eye disease 9; the only treatment option with the surgery leads to the poor sight recovery [10, 11]. Therefore, it is urgent to develop a pharmacological approach for the therapy of PVR.

In the PDGFR family the products of two genes *PDGFRA* and *PDGFRB* can form three dimers: PDGFRαα, PDGFRαβ and PDGFRββ, which are receptor tyrosine kinases (RTKs) [12]. There are activated PDGFRα and PDGFRβ in ERMs from PVR patients [13], and in the ERMs there are a variety of cell types including RPE cells, glial cells, fibroblasts and macrophages [8, 14]. The PDGF family contains five protein members: PDGF-AA, -BB, -AB, -CC and -DD, which are produced by four genes: *PDGFA*, *PDGFB*, *PDGFC* and *PDGFD*, respectively. Notably, while PDGF-AA is a specific ligand for PDGFRαα, PDGF-BB can bind to all the PDGFR dimers: PDGFRαα, -αβ and -ββ. In addition, PDGF-CC resembling PDGF-AB can bind to PDGFRαα and -αβ, whereas PDGF-DD can bind to PDGFRββ with a strong affinity, but to PDGFRαβ with a weak affinity [15, 16].

Traditionally, binding of a ligand (e.g., PDGF) specifically to a RTK (e.g., PDGFR) induces the RTK’s dimerization and conformation change, leading to activation of its intracellular kinase domain that phosphorylates itself at a number of tyrosine sites. Subsequently, the phosphorylated tyrosine can be bound by intracellular enzymes with a Src homology (SH)2 domain, for instance, Src, and adaptors including a p85 regulatory subunit of phosphorinositide 3-kinases (PI3K) [16–18]. As a consequence, the extracellular signal is transduced to a variety of intracellular enzymatic activation including the signaling pathway of PI3K/Akt. As a serine and threonine kinase, Akt plays an essential role in numerous cellular events including cell growth, survival, dedifferentiation, motility, and metabolism [19, 20]. Up-regulation of Akt activity is strongly associated with a number of human diseases including cancer [17, 21] and PVR [22–25].

In addition, PDGFRα could be activated indirectly via an intracellular route of reactive oxygen species (ROS) and Src family kinases (SFKs) [24, 26]. Here, ROS comes from Rac GTPases–regulated nicotinamide adenine dinucleotide phosphate (NADPH) oxidase [27] and mitochondria due to the decreased autophagy [25]. That is, growth factors outside the PDGF family (non-PDGFs) are able to activate the pathway of PDGFRα/PI3K/Akt/mTORC1 (mammalian target of rapamycin complex 1), resulting in a reduction in autophagy [25]. This module of action has been demonstrated using fibroblasts derived from genetically modified mouse embryos 25, 28; that is, indirect activation of PDGFRα via an intracellular route of ROS/SFKs contributes to the development of PVR [23, 29].

RPE cells play a critical role in the PVR pathogenesis because these cells are the major component of ERMs from patients with PVR [8, 14]. Thanks to the advent of the technology of the clustered regularly interspaced short palindromic repeats (CRISPR)-associated endonuclease (Cas)9 [6, 30–32], we recent discovered that PDGFRβ is the predominant isoform among the PDGFRs in RPEM cells, which were the RPE cells derived from ERMs from patients with PVR [16, 33, 34], and that PDGFRβ plays an essential role in vitreous-induced activation of Akt in the RPEM cells and cellular events related to the PVR pathogenesis [16]. Thereby, we hypothesized that in RPE cells non-PDGFs in the vitreous were able to activate PDGFRβ indirectly via an intracellular pathway of ROS/SFKs, and a truncated PDGFRβ lacking a PDGF binding domain [35, 36] was harnessed to test this hypothesis.

## Materials and methods

### Major reagents and Cell Culture

Antibodies against p-Akt (p-S473) (Catalog #: 9271), Akt (Catalog #: 9272), p-PDGFRβ (p-Y751, catalog #: 3166) and PDGFRβ (Catalog #: 3162) were purchased from Cell Signaling Technology (Danvers, MA), an antibody against α-smooth muscle actin (α-SMA) Catalog #: ab5694) was from Abcam (Danvers, MA), a heat shock protein (Hsp)90α (Catalog #: PA3-0137) was from ABR Affinity Bioreagents (Golden, CO), and a β-actin antibody (Catalog #: sc-47,778) was purchased from Santa Cruz Biotechnology (Santa Cruz, CA). HRP (horseradish peroxidase)-conjugated goat anti-rabbit IgG (Catalog #: sc-2004) and goat anti-mouse IgG (Catalog #: sc-2005) secondary antibodies were ordered from Santa Cruz Biotechnology. Enhanced chemiluminescent substrate for detection of horseradish peroxidase was from Thermo Fisher Scientific (Waltham, MA). N-acetyl-cysteine (NAC, a scavenger of ROS) and SU6656 (a selective inhibitor of Src family kinases) [23] were purchased from Sigma (St. Luis, MO) and Calbiochem (San Diego, CA), respectively.

Normal rabbit vitreous (RV) was prepared by dissection from normal rabbit eyeballs when there were still frozen, and the thawed vitreous was centrifuged at 4 °C for 5 min (min) at 10,000 ×g. The resulting supernatant was used for all analyses. There is hardly detectable PDGF in RV as demonstrated previously [23].

RPEM cells were derived from an epiretinal membrane from a patient with grade C PVR and expressed RPE-cells’ markers including keratin as described previously [16]. These RPEM cells were gifts from Dr. Joanne Matsubara at the University of British Columbia, Canada, and grown in Dulbecco’s modified Eagle’s medium/nutrient mixture (DMEM/F12, Thermo Fisher Scientific) supplemented with 10% fetal bovine serum (FBS) and antibiotics of streptomycin (50 ug/ml) and penicillin (50 units/ml). When these cells were sub-cultured, 1:2 split was performed in their 90% confluence.

Human embryonic kidney (HEK) 293GPG cells were a gift from the Kazlauskas lab at the Schepens Eye Research Institute (Boston, MA) and were grown in high-glucose (4.5 g/L) DMEM supplemented with 10% FBS, G418 (0.3 mg/ml), tetracycline (1 ug/ml) and puromycin (2 ug/ml). These 293GPG cells were stably transfected with genes of vesicular stomatitis virus including the *gag*, *pol*, and the *VSV-G* gene. All mammalian cells were cultured at 37 °C in a humidified incubator with 5% CO_2_ [37].

### Construction of PDGFRβ∆x

Construction of PDGFRβ∆x was completed in two steps. First, the PDGFRβ∆x lacking amino acids 38 to 442 of the human PDGFRβ was cloned into the PVZ-*ApaI-NotI-EcoRI-XbaI-SalI-PstI-HindII* vector [35] using *EcoRI/Xbal*. Then the digested *PDGFRβ∆x* DNA fragment with *EcoRI/SalI* was subcloned into *pLXSHD*-*EcoRI-HpaI-XhoI-BamHI* vector digested with *EcoRI/Xhol*. The resulting construct was termed *pLXSHD*-*PDGFRβ∆x* and verified by Sanger DNA sequencing at the MGH DNA core facility (Cambridge, MA).

### Production of retrovirus

In the production of retrovirus there were three steps: transfection, collection of retrovirus, and concentration. Transfection: Gently mix lipofectamine 2000 (156 µl, Thermo Fisher Scientific ) *pLXSHD* or *pLXSHD*-*PDGFRβ∆x* (25 µg) in an OPTIMEM medium (1.8 ml, Thermo Fisher Scientific), incubate these mixtures at room temperature for 30 min so as to form liposomes entraping the DNA, and then transfer these liposomes dropwise into the 293GPG cells, which were in about 70% confluence in a 15-cm cell culture dish. Notably, during transfection, the growth medium was changed to 10 ml OPTIMEM, and after transfection for 7–10 h, a 12 ml virus-producing medium (high glucose DMEM with 10% FBS) was added. In the following morning, a 20 ml fresh virus-producing medium was used to replace the old medium.

Collection of retrovirus: harvest the culture media containing retroviruses after transfection at 48, 72, 96, 120 h, and spin at 1500 rpm for 10 min to remove cells and debris.

Concentration: Spin the supernatant containing the virus at 25, 000 ×g, 4 °C for 90 min, dissolve the white pellet on the bottom of the centrifuge tube in 300 µl of sterile TNE buffer (50 mM Tris pH 7.8, 130mM NaCl, 1 mM EDTA), and then gently rotate the solution overnight at 4 °C to obtain the retrovirus [38].

### Expression of PDGFRβ∆x in RPEM cells

Depletion of PDGFRβ in RPEM cells was achieved using the CRISPR/Cas9 technology with a PB3 sgRNA (GCCTGGTCGTCACACCCCC) guiding SpCas9 to cleave human genomic *PDGFRB* at exon 3 as described previously [16]. These PDGFRβ-depleted RPEM cells were infected by the concentrated retrovirus in DMEM supplemented with 10% FBS and 8 µg/mL polybrene (hexadimethrine bromide; Sigma, St. Louis, MO). Cells expressing PDGFRβ∆x were selected in a histidine-free DMEM supplemented with 2 mM L-histidinol dihydrochoride (Sigma), and the levels of the PDGFRβ∆x in these cells were determined by western blot with an anti–PDGFRβ antibody recognizing the intracellular domain of PDGFRβ [16].

### Western blot

As described previously [16], proteins in cell lysates after centrifugal clarification at 13,000 ×g for 10 min were mixed with sample buffer and denatured by boiling for 5 min. Subsequently the soluble proteins in the sample buffer were separated by 10% SDS-polyacrylamide gel electrophoresis. The proteins in the gel were then transferred to polyvinylidene difluoride membranes for western blot analysis with desired antibodies [16].

### Cell proliferation assay

RPEM cells after trypsin detachment were counted and seeded into wells of a 24-well plate in DMEM/F12 with 10% FBS at a density of 3 × 10^4^ cells/well. After attaching the plate, the RPEM cells were treated with DMEM/F12 only or RV (1:3 dilution in DMEM/F12) with additional NAC (5 mM), SU6656 (1 µM) or their solvent. The cells were trypsin detached after treatment for 48 h for cell counting. Each experimental condition was treated in duplicate, and data from three independent experiments were subjected to statistic analysis [26, 29].

### A scratch wound assay

When RPEM cells grew to near confluence in wells of a 24-well plate, the wells were scratched with a 200 µl pipet tip. After washing with phosphate-buffered solution (PBS), the cells were treated with a medium of DMEM/F12 only or RV (1:3 dilution in DMEM/F12) with or without addition of NAC (5 mM) or SU6656 (1 µM). 16 h later the wound areas were photographed and analyzed with Adobe Photoshop CS6 software. Data from three independent experiments were subjected to statistic analysis [16, 39].

### Contraction assay

RPEM cells were trypsin detached, counted and mixed with collagen I (INAMED, Fremont, CA) on ice. The final collagen I concentration was 1.5 mg/ml, cell density was at 1 × 10^6^ cells/ml, and the pH value was adjusted to 7.2 as described previously [23, 29]. 300 µl cell-gel mixture was transferred into wells of a 24-well plate, which had been preincubated with 5 mg/ml bovine serum albumin/PBS for at least 8 h. 90 min later the collagen gel was polymerized at 37 °C, and 0.5 ml DMEM/F12 or RV (1:3 dilution in DMEM/F12) with or without additional NAC (5 mM) or SU6656 (1 µM). The gel diameter was measured and photographed on day 2 or 3 for further analysis. Data from three independent experiments were subjected to statistic analysis [16, 29, 40].

### Statistics

As described previously [8, 16], data were collected from 3 independent experiments for analysis using ordinary one-way analysis of variance (ANOVA) followed by Tukey’s honest significant difference (HSD) post hoc test. A significant difference between groups was determined by a *P* value less than 0.05.

## Results

PDGFRβ in the RPE cells plays a critical role in vitreous-induced activation of Akt and cellular responses intrinsic to the pathogenesis of PVR [16], but whether non-PDGFs in the vitreous play a part in these signaling and cellular events was unknown. Our original goal was to answer this intriguing question, and our hypothesis was that non-PDGFs were able to indirectly activate PDGFRβ via an intracellular pathway of ROS/SFKs based previous findings [26]. To test this hypothesis, we employed a truncated PDGFRβ lacking a PDGF binding domain [35, 36] and specific inhibitors of ROS and SFKs. The experiments showed that non-PDGFs in the vitreous could activate PDGFRβ and Akt via the intracellular domain of PDGFRβ, leading to cellular responses of proliferation, EMT, migration and contraction related to the development of PVR.

### Expression of a truncated PDGFRβ lacking a PDGF binding domain in the *PDGFRB*-silent RPE cells restores vitreous-induced Akt activation

**Fig. 1:**
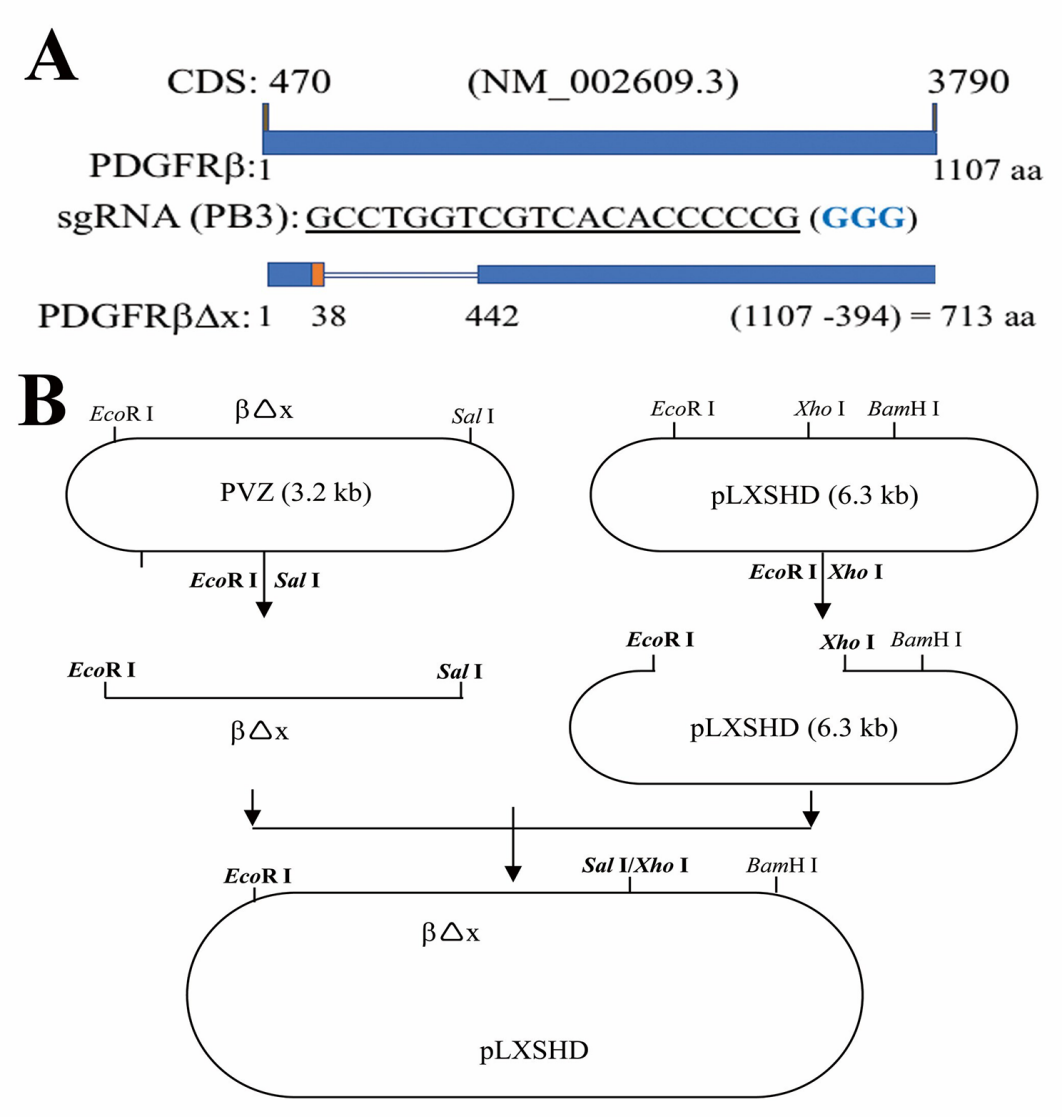
Construction of a retroviral vector to express a truncated PDGFRβ. **(A)** The coding sequence (CDS) of human *PDGFRB* from nucleotide 470 to nucleotide 3790 (NM_002609.3) codes human PDGFRβ: 1107 amino acids (aa) [46]. The protospacer GCCTGGTCGTCACACCCCCG (563–583) from human *PDGFRB* exon 3 was used to generate a specific guide RNA (PB3-sgRNA), which guided *Streptococcus pyogenes* (Sp) Cas9 to cleave human genomic *PDGFRB* in RPE cells, resulting in depletion of PDGFRβ. The blue colored three nucleotides GGG is protospacer adjacent motif (PAM) for SpCas9 recognition [16]. The truncated human PDGFRβ lacking the 38th-442th amino acids of its wild type PDGFRβ was denoted as PDGFRβ∆x and its coding sequence was not targeted by PB3-sgRNA. **(B)** The coding sequence of PDGFRβ∆x was subcloned from a PVZ vector [35] to a *pLXSHD* retroviral vector

**Fig. 2:**
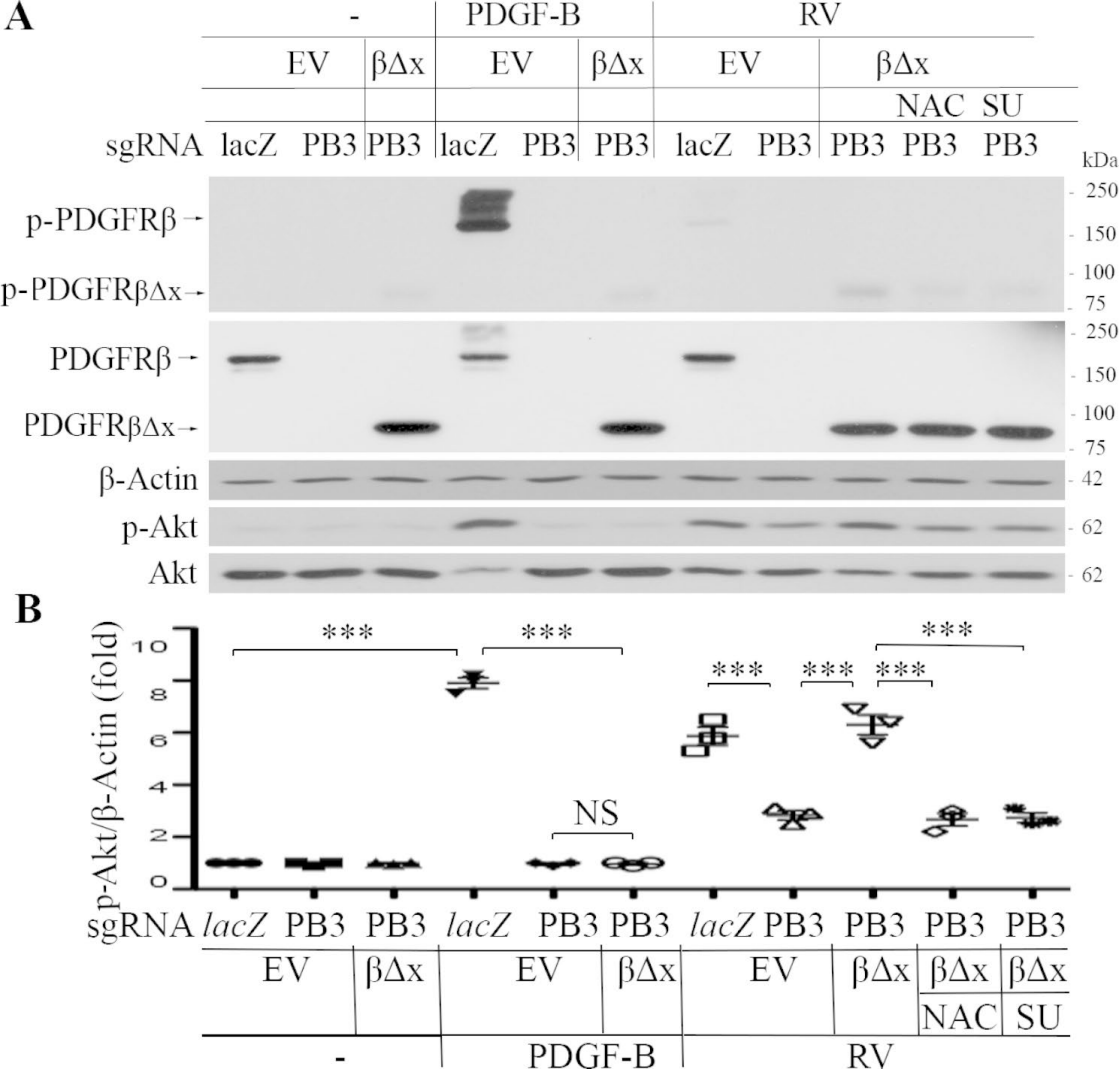
Vitreous induces Akt activation via the truncated PDGFRβ. **(A)** Representative images of western blot with indicated antibodies. When RPEM cells grew to 80% confluence, they were serum-starved overnight and then treated with RV, PDGF-B or RV in the presence of NAC or SU6656 for 15 min. The resultant lysates were subjected to western blot using antibodies against p-PDGFRβ, PDGFRβ, p-Akt, Akt and β-actin. sgRNA-*lacZ* as a control for sgRNA-PB3 (targeting human *PDGFRB*), RV (rabbit vitreous) diluted (1:3) in the medium of DMEM/F12, PDGF-B (10 ng/ml), NAC: N-acetyl-cysteine (5 mM), SU: SU6656 (1 µM), a specific inhibitor of SFKs. EV: empty vector (*pLXSHD*), β∆x: a truncated PDGFRβ lacking the PDGF-binding domain. **(B)** The intensity of the p-Akt bands in A was first normalized to that of the corresponding β-actin bands and then calculated to establish the ratio of the control in the first lane, shown as “Fold”. The mean ± standard deviation (SD) of 3 independent experiments is shown; *** denotes p<0.001, and NS stands for not significant using ANOVA

To seek an answer to the question whether non-PDGFs in the vitreous activated PDGFRβ via an intracellular pathway in the RPE cells, a truncated PDGFRβ that does not have a PDGF-binding domain that spans from 38th to 442th amino acids of PDGFRβ [35] was expressed in the RPE cells whose *PDGFRB* had been silent using the CRISPR/Cas9 technology [16]. The single guide (sg) RNA denoted as PB3 guiding SpCas9 to specifically edit genomic *PDGFRB* could not recognize the mutant *PDGFRB* encoding the truncated PDGFRβ (Fig. 1). In this CRISPR/Cas9 system, the PB3-sgRNA was designed to target human *PDGFRB* at exon 3, and the three nucleotides (GGG) of the protospacer adjacent motif (PAM) for the PB3-sgRNA were absent from the mutant *PDGFRB* (Fig. 1A). Thereby we constructed a retroviral vector with the *PDGFRB* mutant to express the truncated PDGFRβ. This truncated PDGFRβ was named as PDGFRβ∆x (Fig. 1B). The retrovirus containing DNA sequence coding PDGFRβ∆x was used to infect the *PDGFRB*-silent RPEM cells (Fig. 2). As expected, PDGFRβ∆x was successfully expressed in the PDGFRβ-depleted RPEM cells, and PDGF-BB activated PDGFRβ and Akt in the control RPEM cells, but neither in the *PDGFRB*-silent or in the PDGFRβ∆x-expressed RPEM cells (Fig. 2). However, RV, in which there are no detectable PDGFs as determined previously [26], activated not only PDGFRβ but also PDGFRβ∆x in the PDGFRβ or PDGFRβ∆x -expressed RPEM cells (Fig. 2). Notably, expression of PDGFRβ∆x in the PDGFRβ-depleted RPEM cells restored vitreous-induced activation of Akt (Fig. 2).

To investigate whether ROS-mediated Src family kinases (SFKs) played a part in RV (non-PDGFs) -induced activation of PDGFRβ in RPEM cells as they do for RV-induced PDGFRα activation in fibroblasts [26], we treated the PDGFRβ∆x-expressed RPEM cells with RV in the presence of either a ROS scavenger N-acetyl-cysteine (NAC) or a SFKs-specific inhibitor (SU6656). Previous experiments identified that 5 mM NAC or 1 µM SU6656 could effectively inhibit vitreous-induced Akt activation and PVR-related cellular events without obvious toxicity to RPE cells [23, 25, 26]. As predicted, Western blot analysis showed that both 5 mM NAC and 1 µM SU6656 inhibited RV-induced activation of PDGFRβ∆x and Akt (Fig. 2), suggesting that both ROS and SFKs are required for non-PDGFs-induced activation of PDGFRβ in RPEM cells.

### Expression of PDGFRβ∆x in the PDGFRβ-depleted RPE cells recovers vitreous-induced cell proliferation, EMT, and migration

**Fig. 3:**
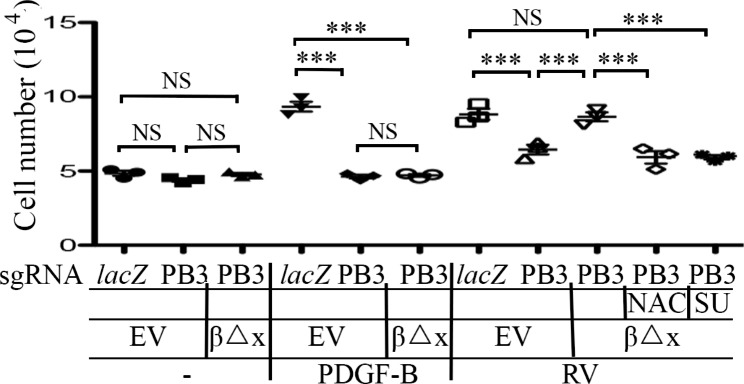
Vitreous stimulates cell proliferation via the truncated PDGFRβ. RPEM cells characterized in Fig. 2 were treated with DMEM/F12, PDGF-B (20 ng/ml) or RV diluted (1:3) in DMEM/F12 in the presence or absence of NAC (5 mM), or SU: (SU6656, 1 µM). After treatment for 48 h the cells were counted with a hemocytometer. The mean ± SD of 3 independent experiments is shown; *** denotes p< 0.001, and NS stands for not significant using ANOVA

Akt activation is essential for a great many of cellular responses including cell proliferation, EMT, and migration [19], which are intrinsic to the PVR pathogenesis [8, 16]. While vitreous stimulation could induce proliferation and migration of RPEM cells [8, 16], PDGFRβ absence from RPEM cells significantly dampens vitreous-induced cellular events [16]. To this end, we investigated if PDGFRβ∆x expression in the *PDGFRB*-silent RPEM cells could resume vitreous-induced cell proliferation and migration. As shown in Fig. 3, while PDGF-BB stimulated RPEM cells’ proliferation, but it failed to do so in those cells expressing PDGFRβ∆x. Nevertheless, vitreous stimulated more proliferation of RPEM cells expressing PDGFRβ∆x than that of those *PDGFRB*-silent cells as shown in Fig. 3. Furthermore, both the ROS scavenger NAC and the SFKs’ inhibitor SU6656 hindered the vitreous-induced cell proliferation of the RPEM cells with PDGFRβ∆x (Fig. 3).

**Fig. 4:**
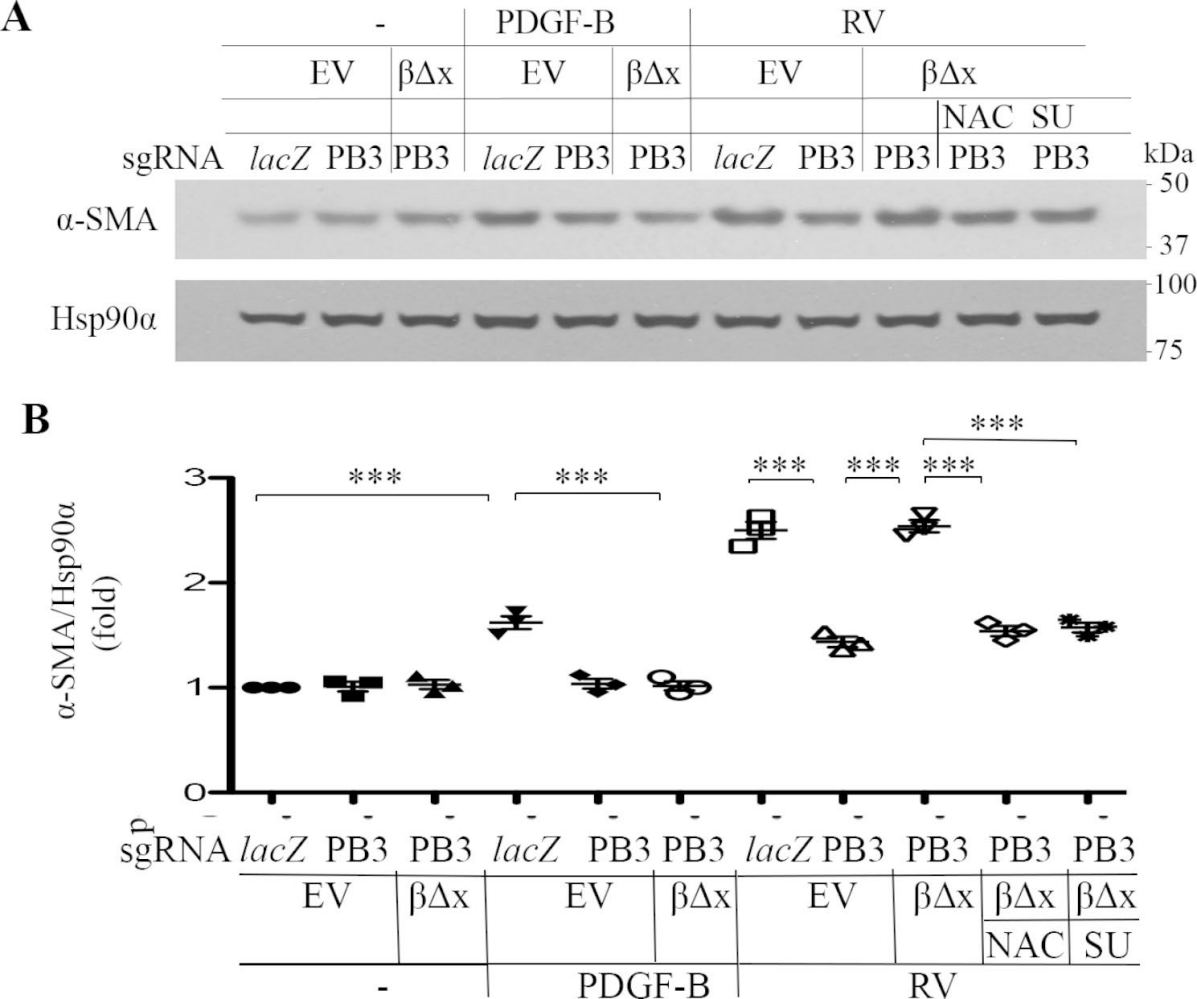
Vitreous stimulates EMT via the truncated PDGFRβ. **(A)** Representative images of western blot with indicated antibodies. α-SMA is a protein marker of EMT, and Hsp90α is a loading control. RV diluted (1:3) in DMEM/F12, NAC: N-acetyl-cysteine (5 mM), SU: SU6656 (1 µM). EV: empty vector, β∆x: a truncated PDGFRβ lacking the PDGF-binding domain. **(B)** The intensity of α-SMA bands in A was first normalized to that of the corresponding Hsp90α bands and then calculated to establish the ratio of the control in the first lane, shown as “Fold”. The mean ± SD of 3 independent experiments is shown; *** denotes p<0.001 using ANOVA

EMT is a critical step in the development of PVR [41]. We next evaluated if suppression of PDGFRβ could blunt RV-induced expression of α-SMA, a protein marker of EMT. As shown in Fig. 4, while RV boosted α-SMA expression, depletion of PDGFRβ in RPEM cells lessened α-SMA expression, whereas introduction of PDGFRβ∆x into these cells restored α-SMA expression induced by RV. Similar to the prior findings, both NAC and SU6656 blocked RV-induced expression of α-SMA in the RPEM cells with PDGFRβ∆x (Fig. 4), suggesting ligand-independent activation of PDGFRβ also plays an important part in RV-induced EMT.

**Fig. 5:**
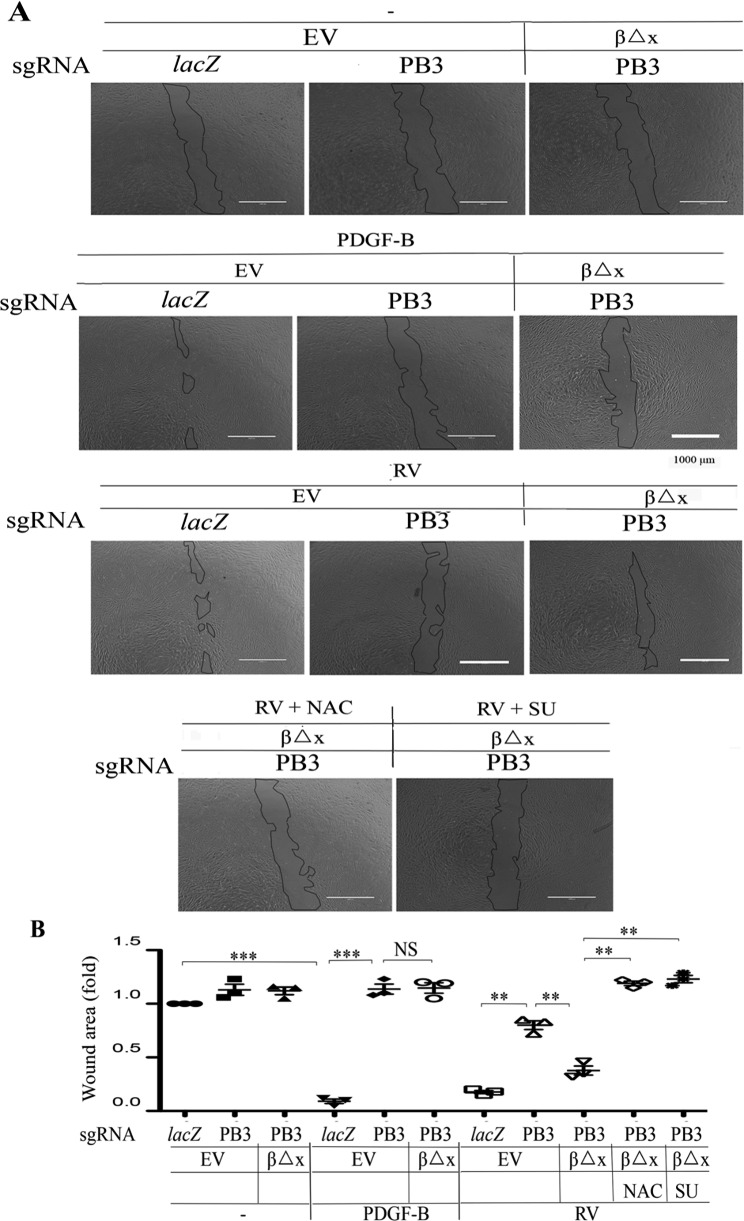
Vitreous stimulates cell migration via the truncated PDGFRβ. **(A)** Confluent RPEM cells described in Fig. 2 were subjected to a scratch wound healing assay. PDGF-B: 20 ng/ml, RV diluted (1:3) in DMEM/F12, NAC: 5 mM, or SU (SU6656, 1 µM). Representative images of cell migration shown were taken after 16 h. Scale bar: 1000 μm. **(B)** Shown is the mean ± SD of 3 independent experiments.** and *** denote p<0.01, p<0.001, respectively, and NS stands for not significant using ANOVA

We next employed a scratch wound assay to estimate the capability of cell migration as previously described [8, 16, 39]. As shown in Fig. 5, PDGF-BB enhanced migration of RPEM cells; however, it did not, but vitreous did, induce more migration of the RPEM cells expressing PDGFRβ∆x than that of those *PDGFRB*-silent cells. As expected, both NAC and SU6656 impeded vitreous-induced migration of PDGFRβ∆x-expressed RPEM cells (Fig. 5).

### Expression of PDGFRβ∆x in the PDGFRβ-depleted RPE cells restores vitreous-induced cell contraction

In the pathogenesis of PVR, ERMs consisting of extracellular matrix and cells including RPE cells contract, resulting in the retinal detachment [8, 14]. To investigate the molecular mechanism of the PVR pathogenesis, an in vitro assay with collagen and RPEMs was established to mimic the in vivo contraction process. In this assay, the characterized RPEM cells in Fig. 2 were firstly mixed with collagen I to form a cell-collagen mixture, analogy to the ERMs. Subsequently, a DMEM/F12 medium with agents (PDGF-BB or RV) in the presence or absence of NAC (5 mM) or SU6656 (1 µM) was added onto the collagen gel. This procedure was to mimic the in vivo environment with a drug treatment.

**Fig. 6:**
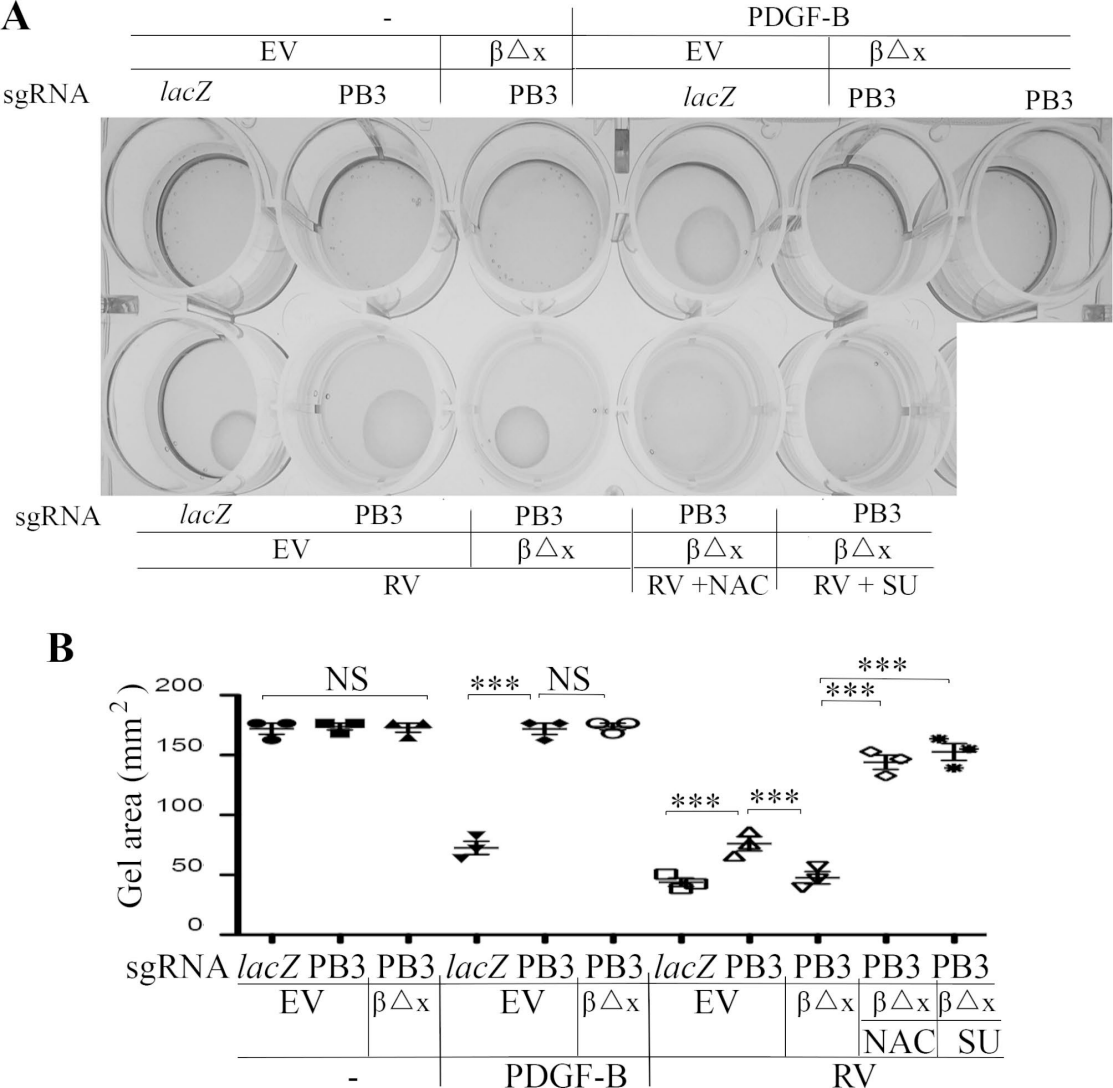
Vitreous induces cell contraction via the truncated PDGFRβ. **(A)** The RPEM cells defined in Fig. 2 were subjected to a collagen contraction assay. DMEM/F12 alone (-), PDGF-B (20 ng/ml) or RV diluted (1:3) in DMEM/F12, NAC: 5 mM, and SU: (SU6656, 1 µM). Shown are images from representative of three independent experiments. **(B)** Shown is the mean ± SD of 3 independent experiments. *** denotes p<0.001, and NS indicates not significant using ANOVA

As shown in Fig. 6, PDGF-BB induced a collagen-gel contraction of the RPEM cells expressing PDGFRβ, but it failed to do so in either *PDGFRB*-silent or PDGFRβ∆x-expressing RPEM cells. In addition, depletion of PDGFRβ suppressed vitreous-stimulated contraction of RPEM cells, whereas expression of PDGFRβ∆x resumed the contractile capability of the engineered RPEM cells (Fig. 6). Furthermore, both NAC (5 mM) and SU6656 (1 µM) blocked vitreous-induced contraction of the PDGFRβ∆x-expressing RPEM cells, demonstrating that expression of PDGFRβ∆x in the *PDGFRB*-silent RPEM cells restored the vitreous-induced cell contraction (Fig. 6), suggesting that vitreous-induced transactivation of PDGFRβ via an intracellular pathway of ROS/SFKs plays a crucial part in PVR-related cellular events.

## Discussion

In this report we have showed that in RPE cells from epiretinal membranes (RPEM cells) activation of PDGFRβ by non-PDGFs in the vitreous via the ROS/SFKs pathway is critical for the PVR-related signaling events (e.g., Akt activation) (Fig. 2) and cellular responses (e.g., proliferation, migration and contraction) (Figs. 3, 4, 5 and 6). In the extracellular domain of PDGFRβ there are 5-Ig-like subdomains (D1-D5). Ligand binding is limited to the D2 and D3 domains, which are missing in the truncated PDGFRβ that we employed to test our hypothesis in this report. Notably, D1 is a small domain containing 3 N-linked glycans, and the membrane proximal D4 and D5 domains play an important part in the PDGFRβ activation [36].

**Fig. 7:**
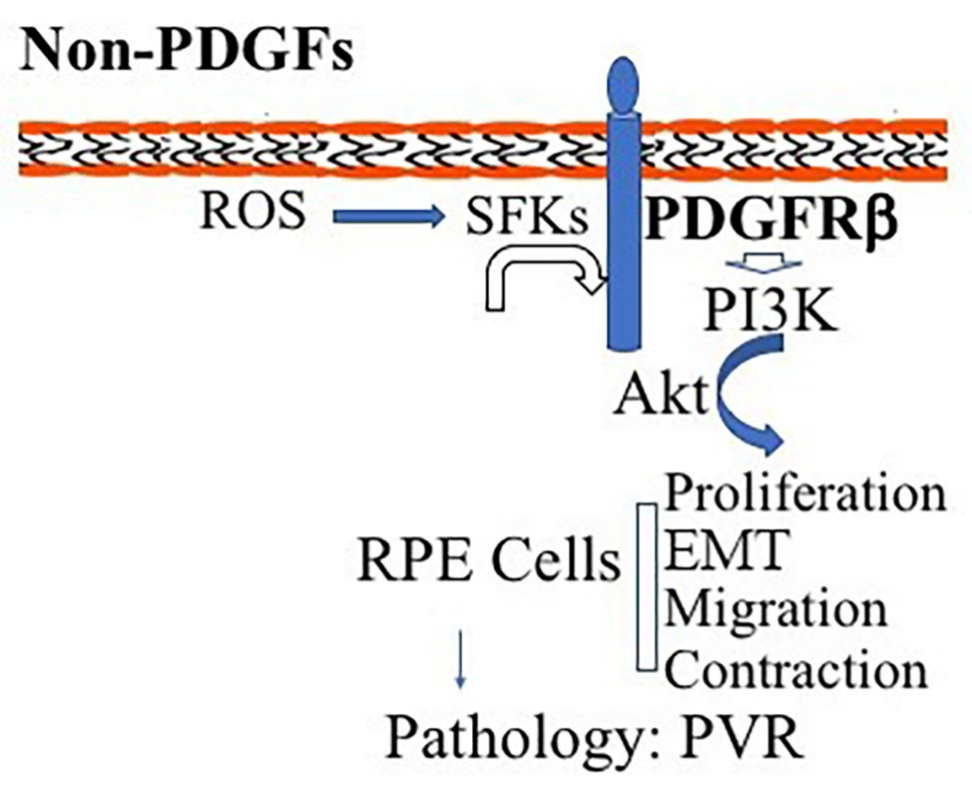
Schematic diagram of non-PDGFs indirectly activating PDGFRβ and its downstream signaling pathway of PI3K/Akt to promote cellular responses intrinsic to PVR pathogenesis. Non-PDGFs (growth factors outside the PDGF family in the vitreous) activate their own receptors, stimulating production of intracellular reactive oxygen species (ROS) that activates Src family kinases (SFKs), which activate PDGFRβ. As a result, the PI3K/Akt signaling is enhanced in retinal pigment epithelial (RPE) cells for promotion of cellular responses including cell proliferation, migration and contraction, leading to the pathogenesis related to proliferative vitreoretinopathy (PVR)

We previously found that in fibroblasts vitreous-induced activation of PDGFRβ intracellularly is hindered by high expression of RasGAP [37], and that in RPE cells expressional levels of RasGAP are lower than those in fibroblasts so that PDGFRβ could play a central role in patient vitreous-stimulated contraction of RPEM cells [16]. In these RPE cells PDGFRα expression is very low so that Akt activation could hardly be induced by PDGF-AA, a PDGFRα-specific ligand [16]. In addition, in RV there is no detectable PDGF 26; thereby we assume that in the PVR pathogenesis the dislocated RPE cells appear in the foreign environment of the normal vitreous in which there are growth factors outside the PDGF family (non-PDGFs) that are able to activate PDGFRβ. This mechanism of PDGFRβ activation in RPE cells is similar to that PDGFRα is activated by non-PDGFs in fibroblasts via the intracellular pathway of ROS/SFKs [23, 25, 26]. In fact, we revealed that both neutralizing ROS by NAC and inhibiting SFKs by SU6656 in RPEM cells attenuated vitreous-stimulating Akt activation as well as cell proliferation, migration and contraction (Figs. 2, 3, 4, 5 and 6). Thereby, we propose that non-PDGFs in the vitreous leads to an increase in intracellular levels of ROS, which activate SFKs, which subsequently somehow activate PDGFRβ. This module of intracellular activation of PDGFRβ is also able to engage its downstream signaling events and cellular responses including proliferation, migration, and contraction (Fig. 7). These extra signaling events and cellular responses are closely linked with the pathogenesis of PVR (Fig. 7).

In the ERMs there are four major cell types: RPE cells, glia cells, fibroblasts and macrophages [42, 43]. The findings reported here that in RPEM cells PDGFRβ is activated indirectly by non-PDGFs in the vitreous, together with our previous discoveries that PDGFRα is activated indirectly in fibroblasts [24–26] place the PDGFR family into the frontier for preventing PVR [1]. The novelty of this report is that not only could the traditional PDGF binding activate PDGFRβ in RPE cells, but also non-PDGFs in the vitreous could indirectly activate PDGFRβ via intracellular mechanisms in which ROS and SFKs play an important part (Figs. 2, 3, 4, 5 and 6). As a matter of fact, indirect activation of PDGFRβ has been reported in other disease models [35, 44, 45].

Notably, the reason we herein used normal rabbit vitreous (RV) instead of human vitreous was because in the RV there is hardly detectable PDGF, whereas in the clinic human vitreous from patients with PVR there are rich PDGFs including PDGF-BB [3], which can activate PDGFRβ, so we can not distinguish the modes of direct (ligand binding) and indirect (non-PDGFs) activation by using patient vitreous. Hence, the RV is a very good source of non-PDGFs, facilitating us to determine if non-PDGFs could activate PDGFRβ indirectly. Nevertheless, there are certain limitations in using RV instead of human vitreous because RV is different from human vitreous and our ultimate goal is to prevent humans from PVR. In addition, the RPEM cells we used in this research were derived from a single ERM from a patient with grade C PVR; thereby our present investigation also has a certain limit as there may be heterogeneity among the cells in ERMs from different patients with PVR.

### Electronic supplementary material

Below is the link to the electronic supplementary material.

Supplementary Material 1

Supplementary Material 2

## Data Availability

All data generated or analyzed during this study are included in this published article and raw data for this article are included in the supplemental file. All data and material in this published article will be available upon a reasonable request for non-commercial purpose.
