# Supplementary material for: Ligand-independent activation of platelet-derived growth factor receptor β promotes vitreous-induced contraction of retinal pigment epithelial cells
Source: BMC Ophthalmol. 2023 Aug 3;23:344. doi: 10.1186/s12886-023-03089-8 (PMC10401781; doi:10.1186/s12886-023-03089-8)

**Supplementary file (raw data)**

**
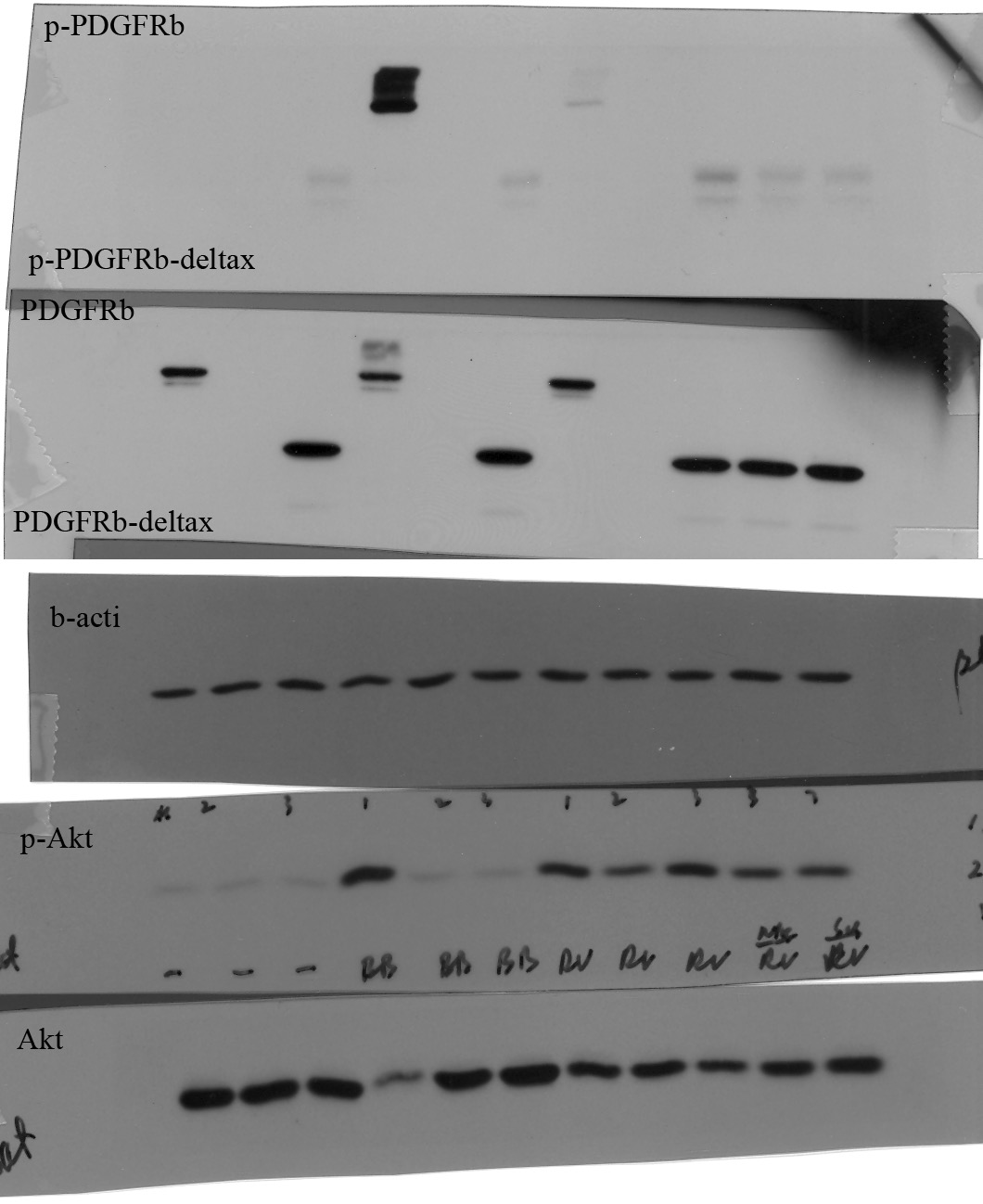
Original Figure 2**

**Original Figure 4**


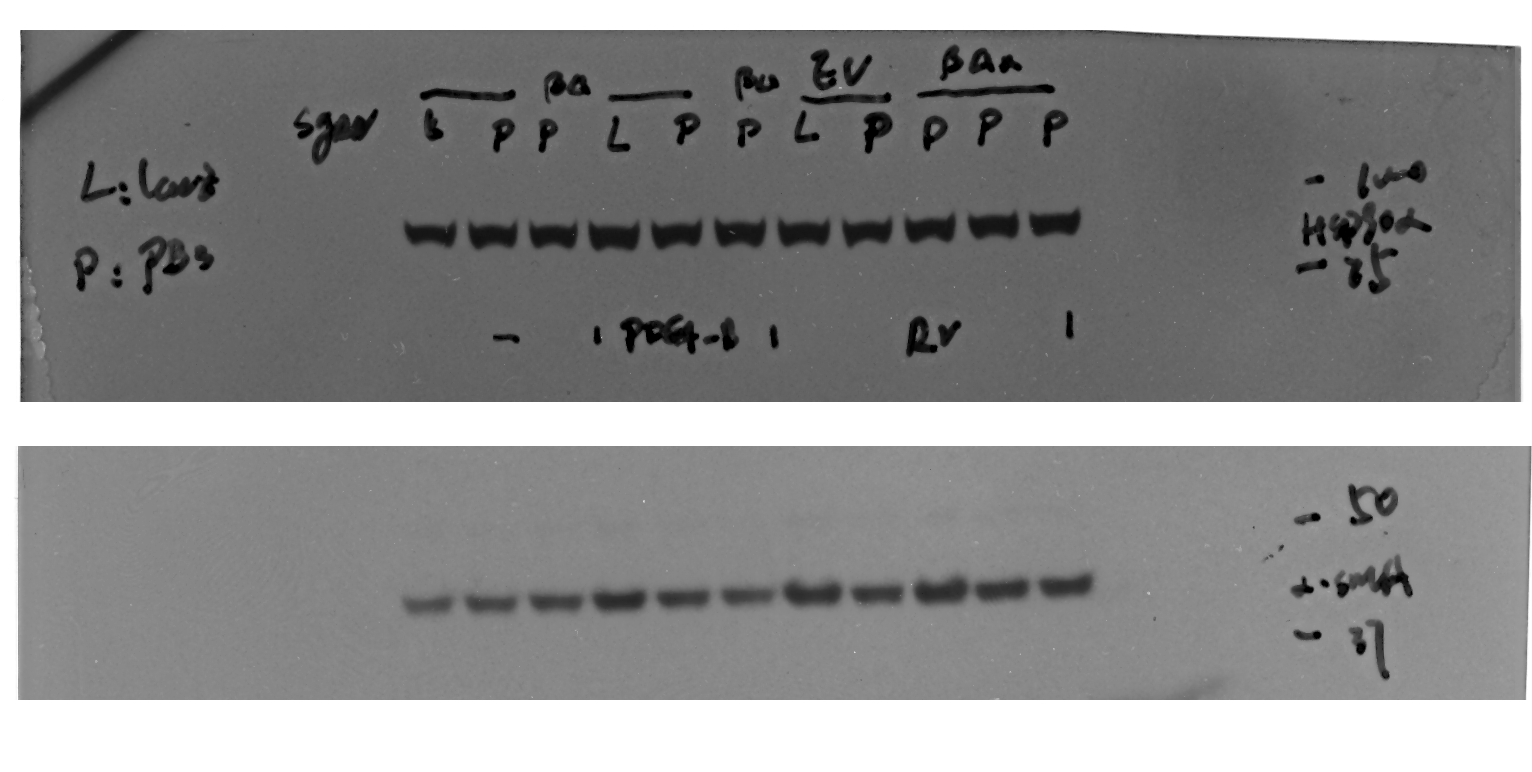


**Original Figure 5**

**Original Fig. 5 (LacZ –EV)**


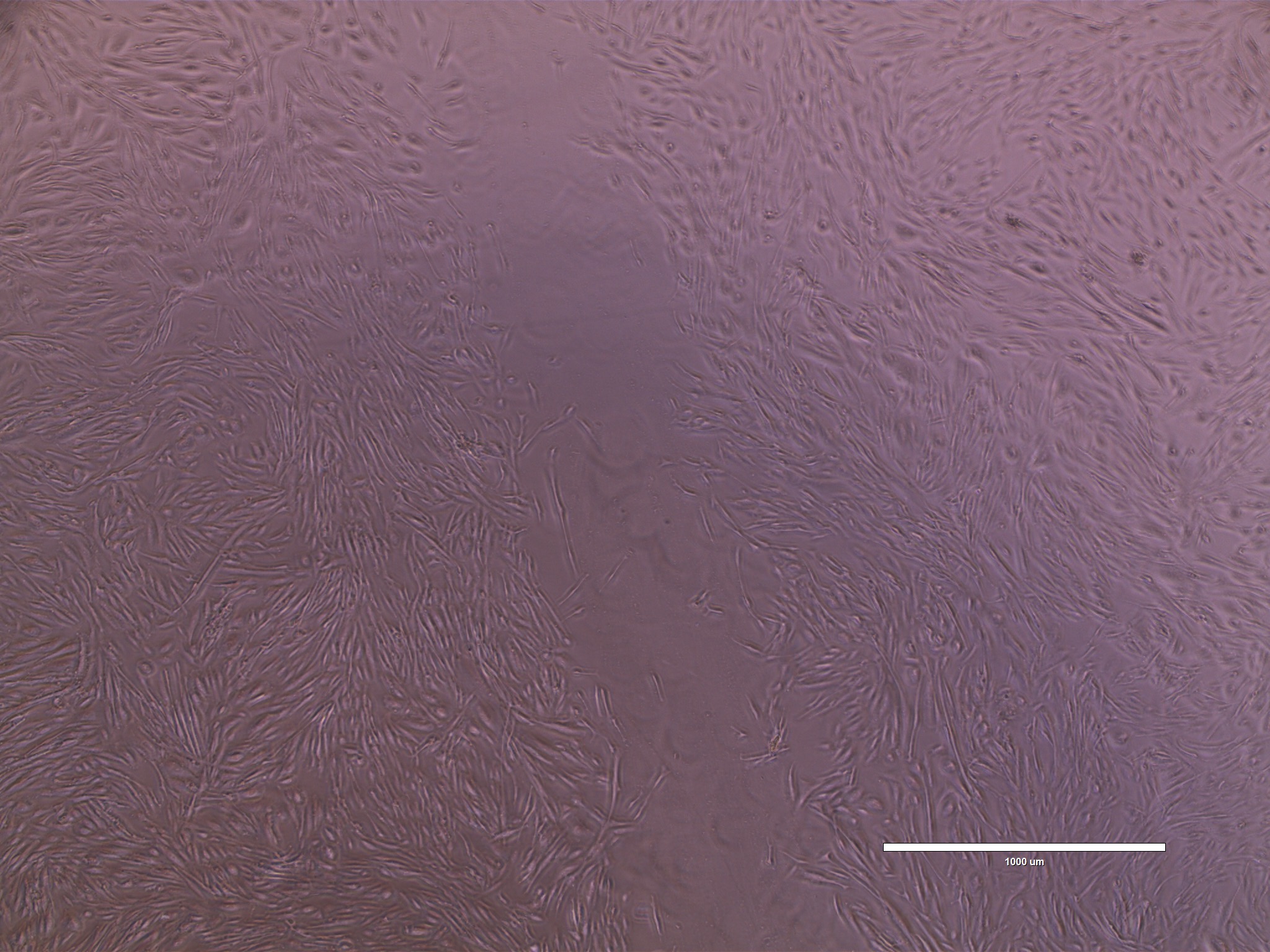


**Original Fig. 5 (LacZ-EV-PDGF-BB)**


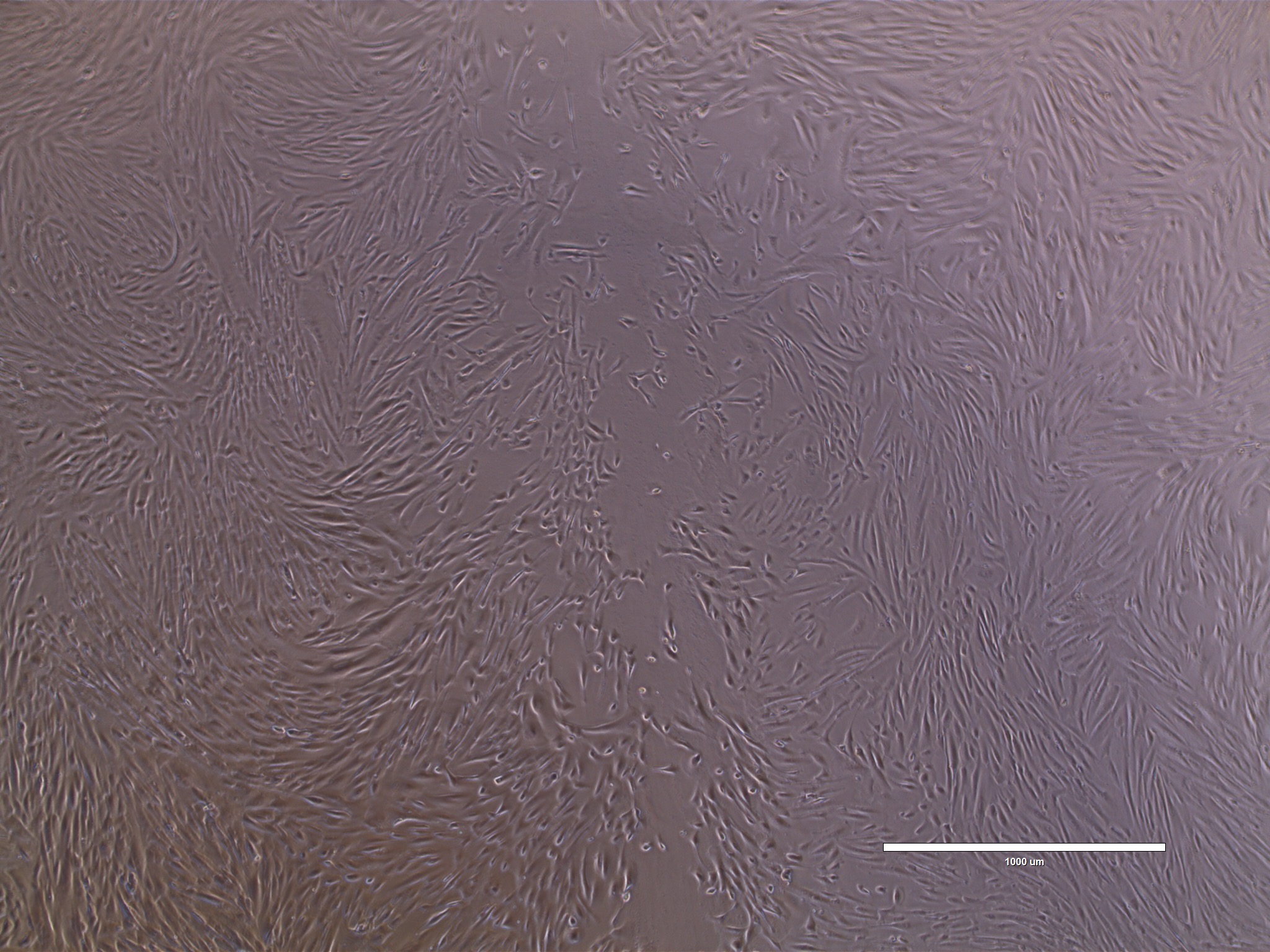


**Original Fig 5 (lacZ-EV-RV)**


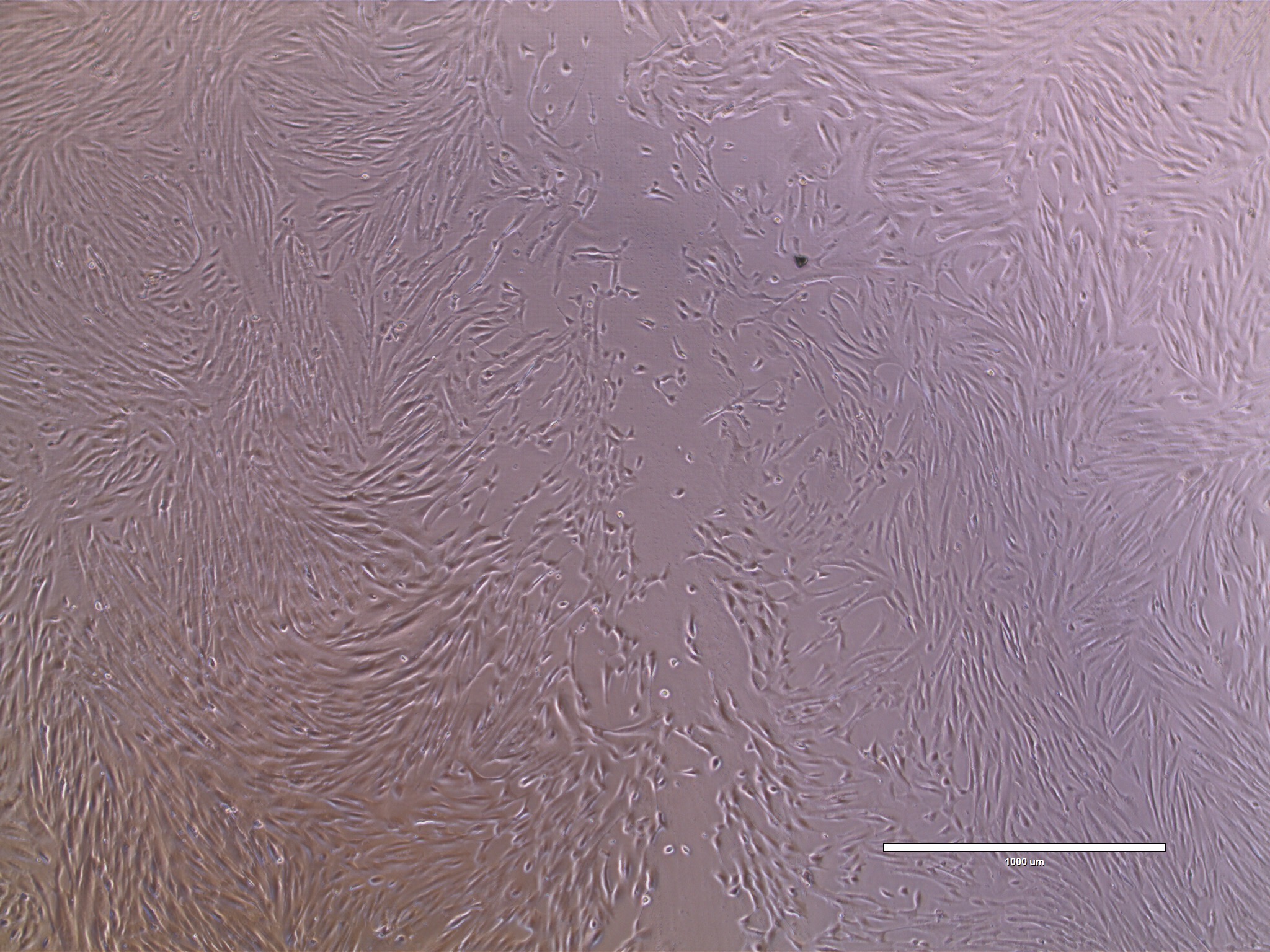


**Original Fig 5 (PB3-EV-PDGF-BB)**


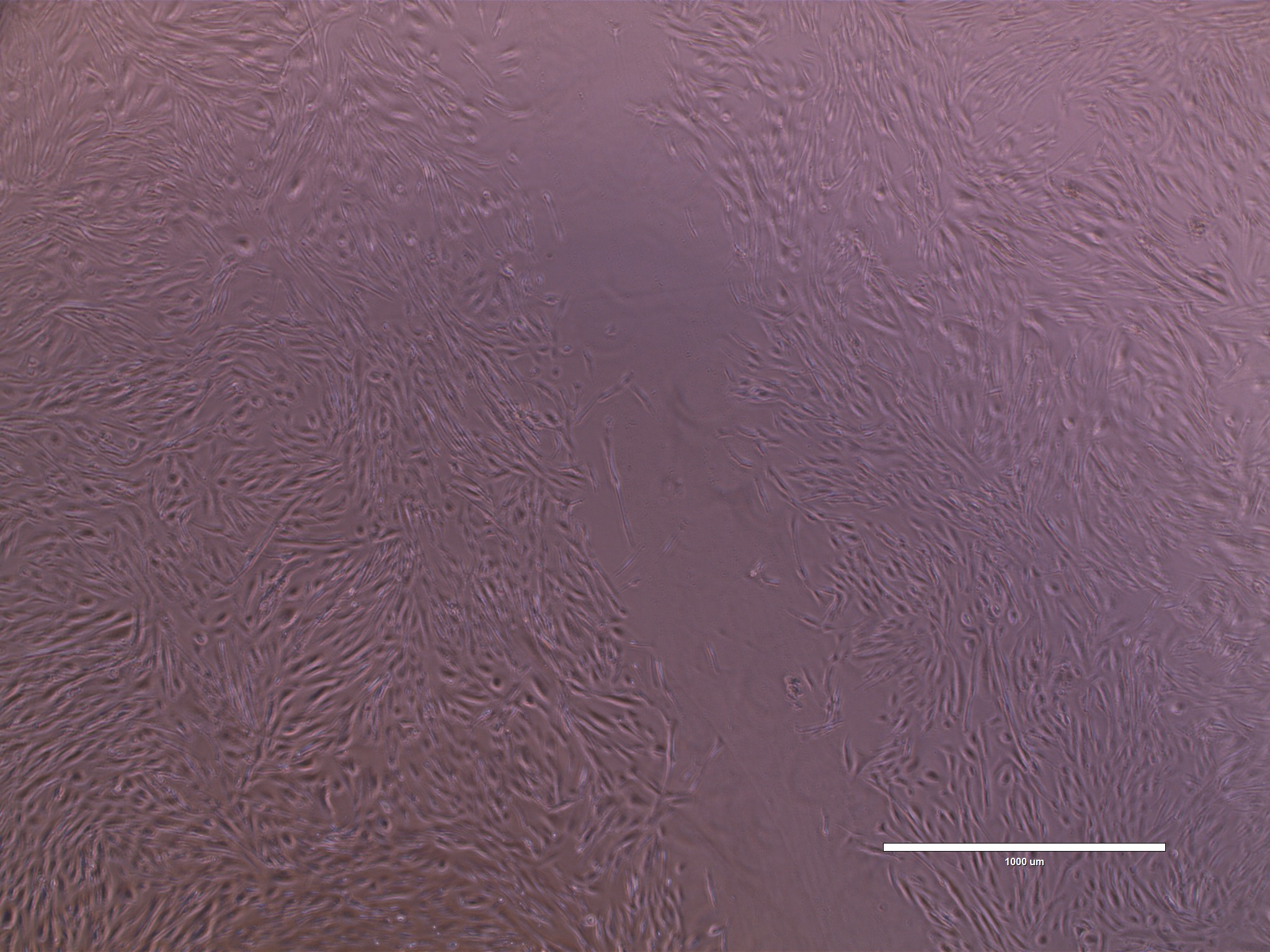


**Original Fig. 5 (PB3-EV-RV)**


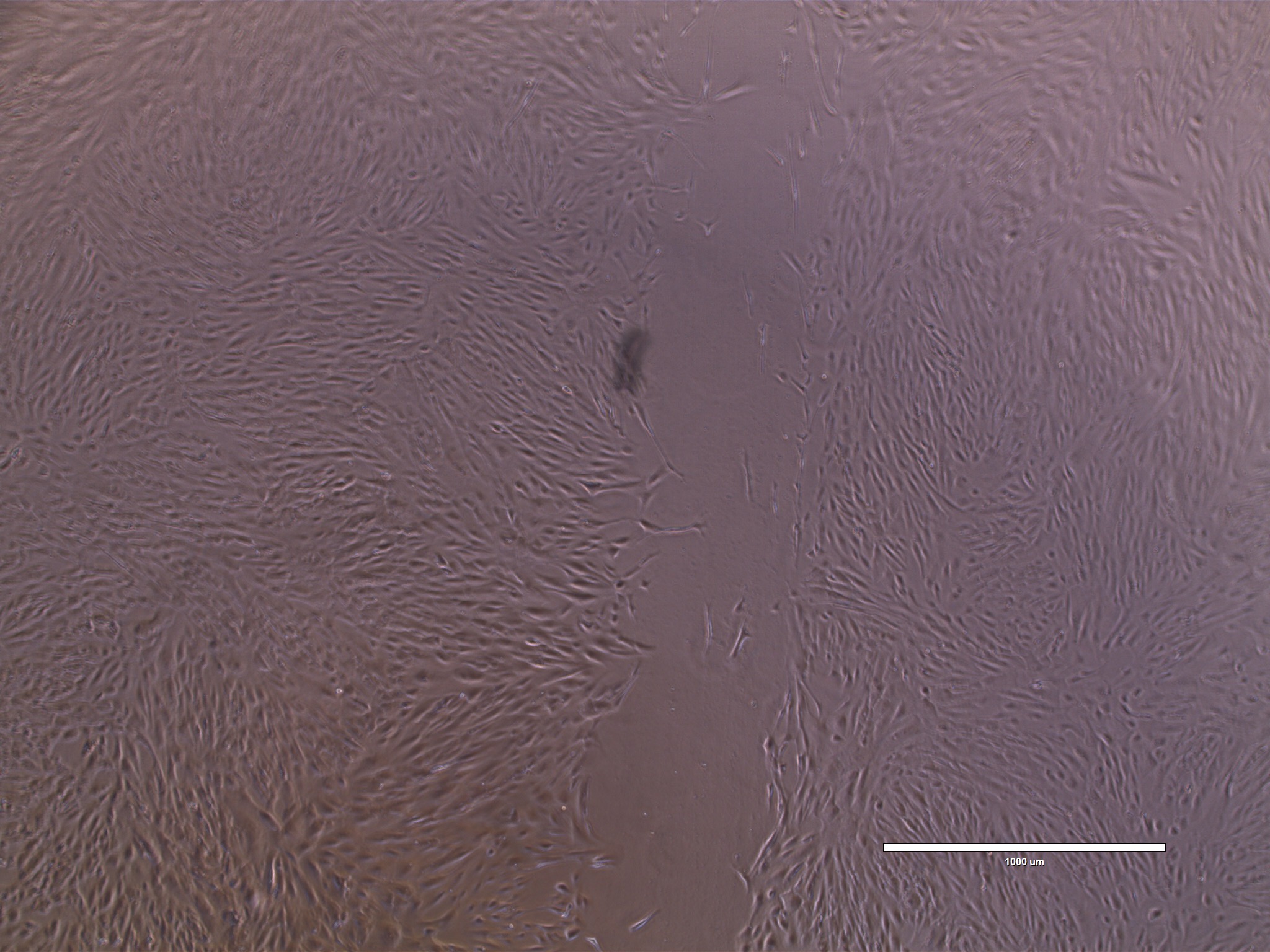


**Original Fig. 5 (PB3-EV)**


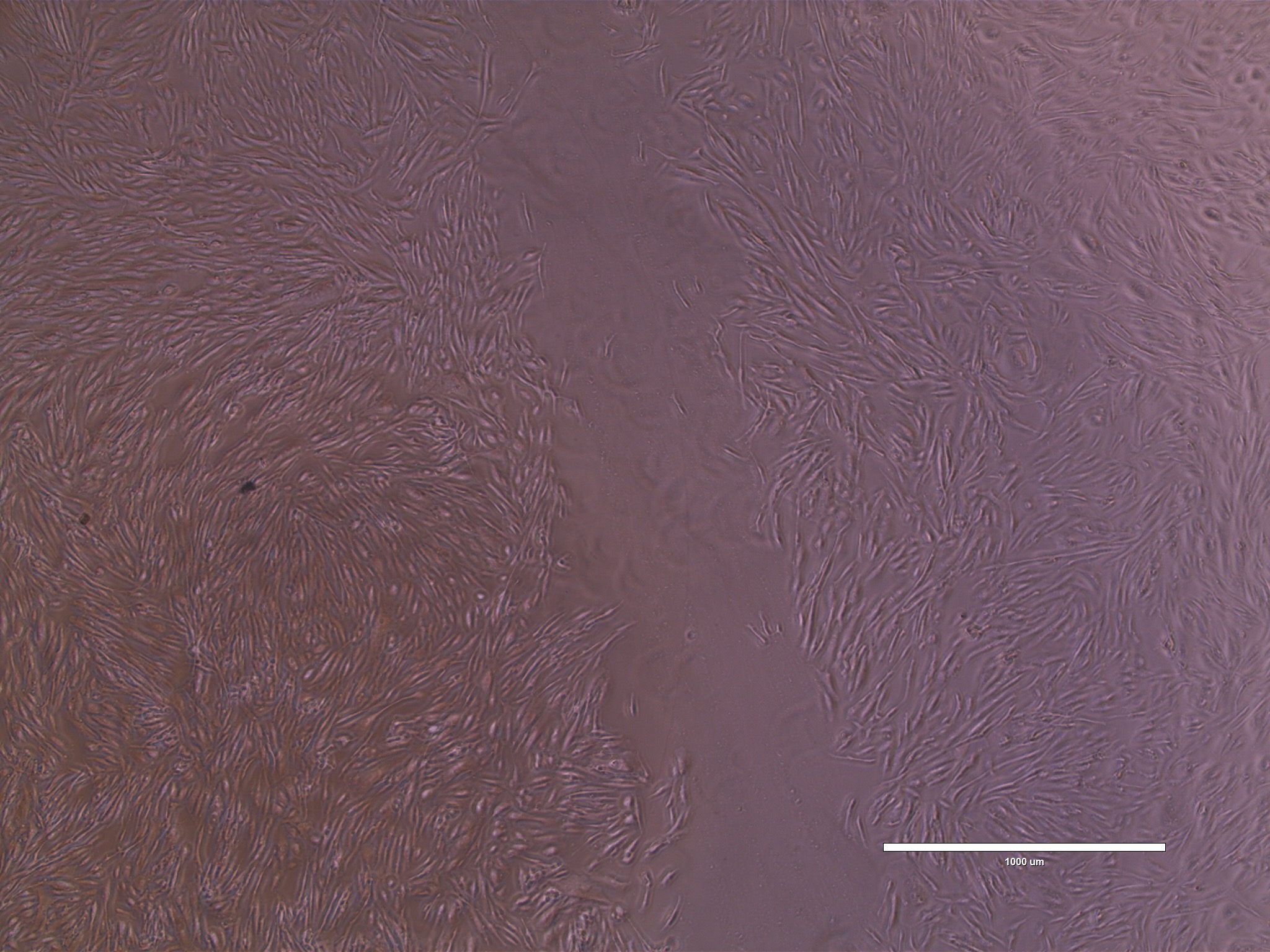


**Original Fig. 5 (PB3-PDGFRB-belta-deltaX)**


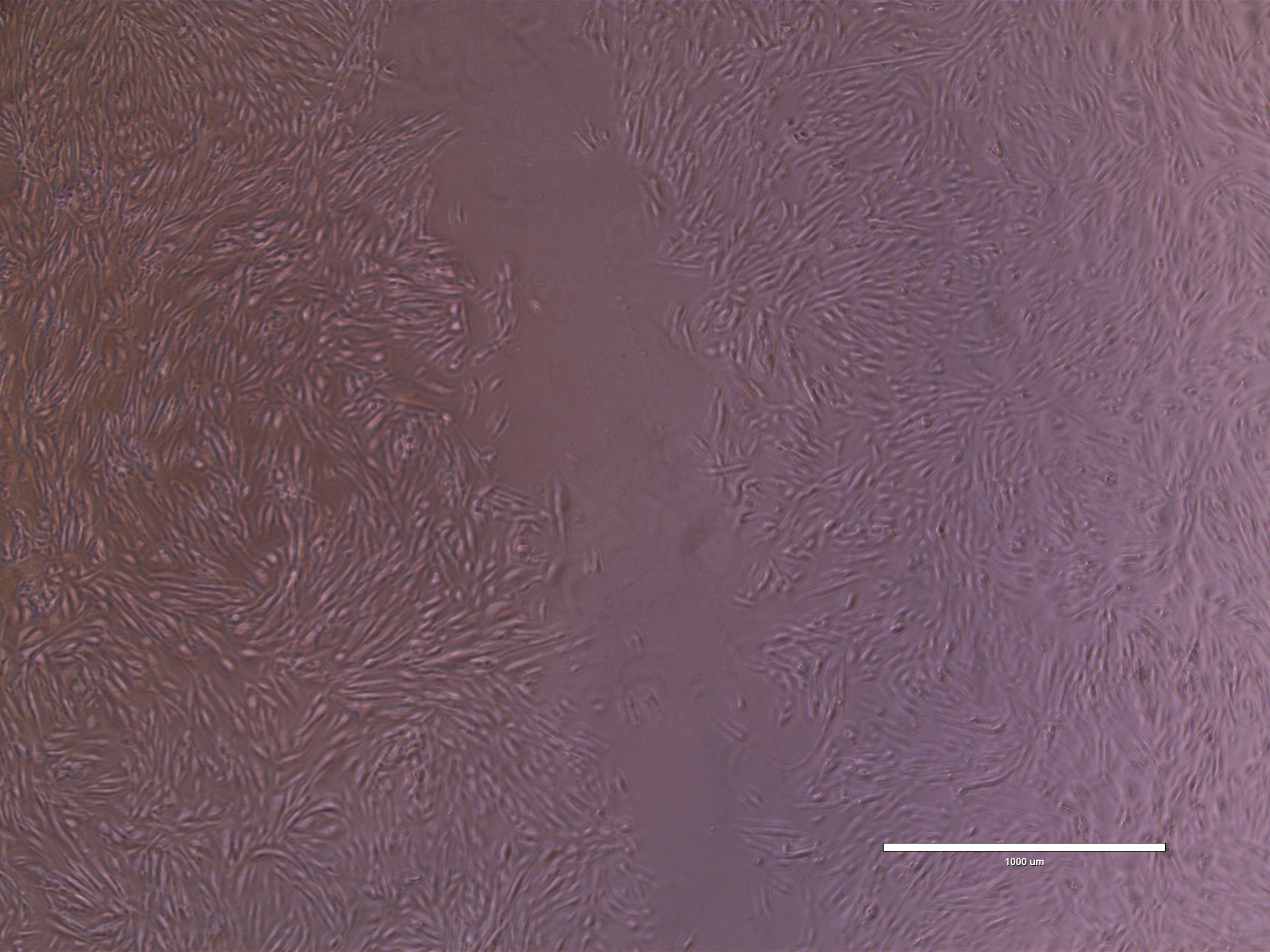


**Original Fig 5 (PB3-bdx-RV)**


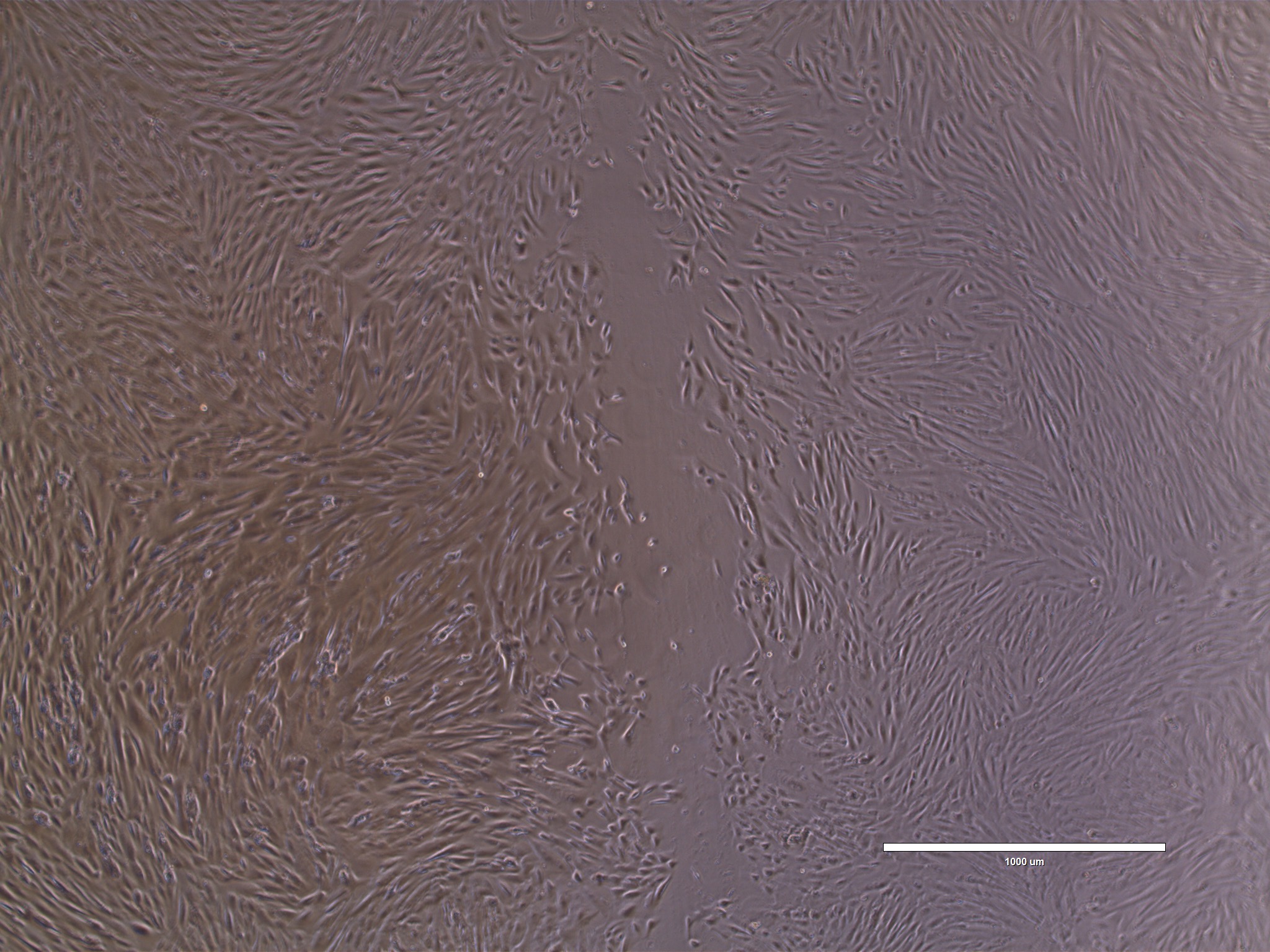


**Original Fig 5 (PB3-bdx-RV-NAC)**


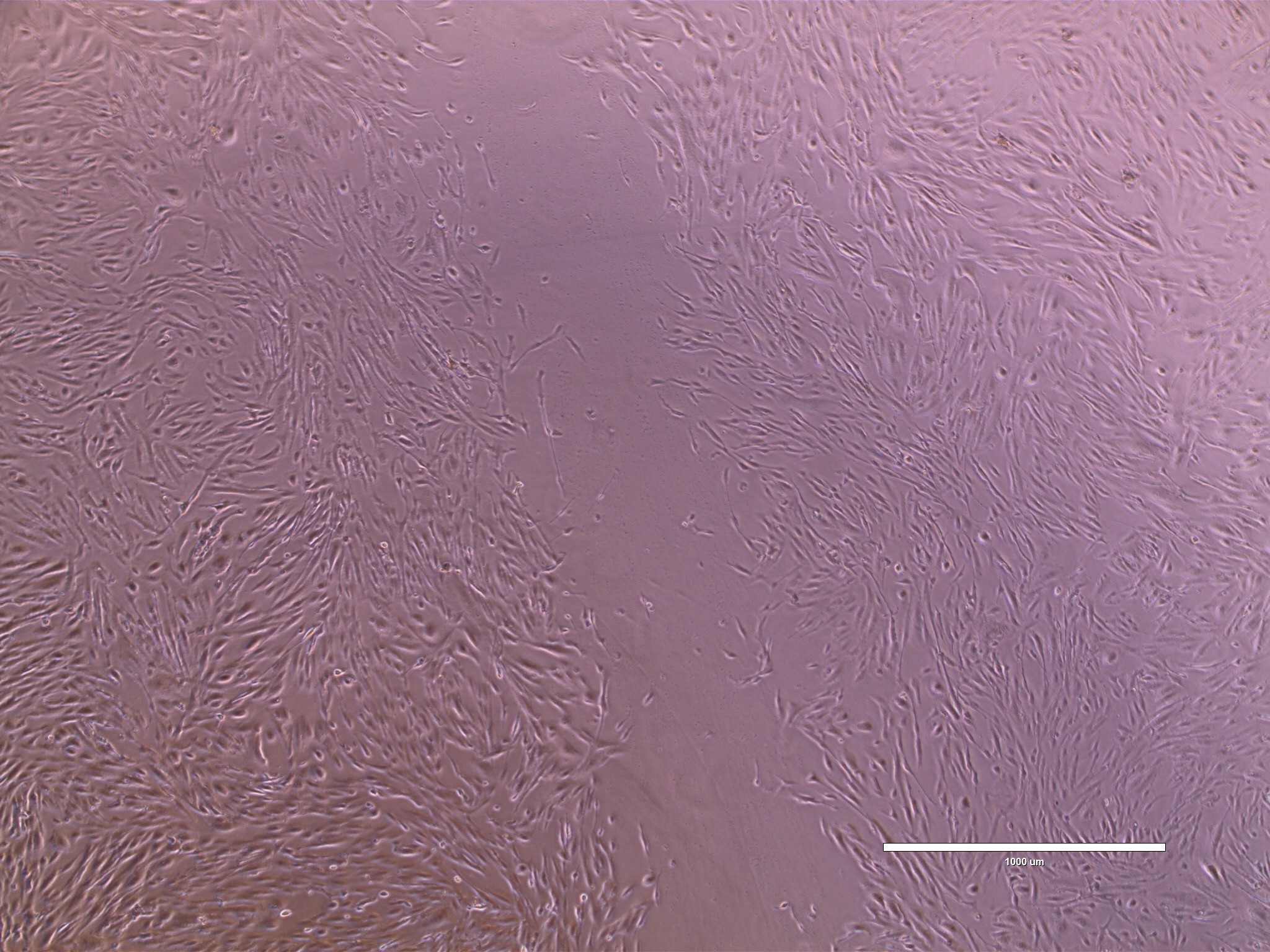


**Original Fig 5 (PB3-bdx-RV-SU)**


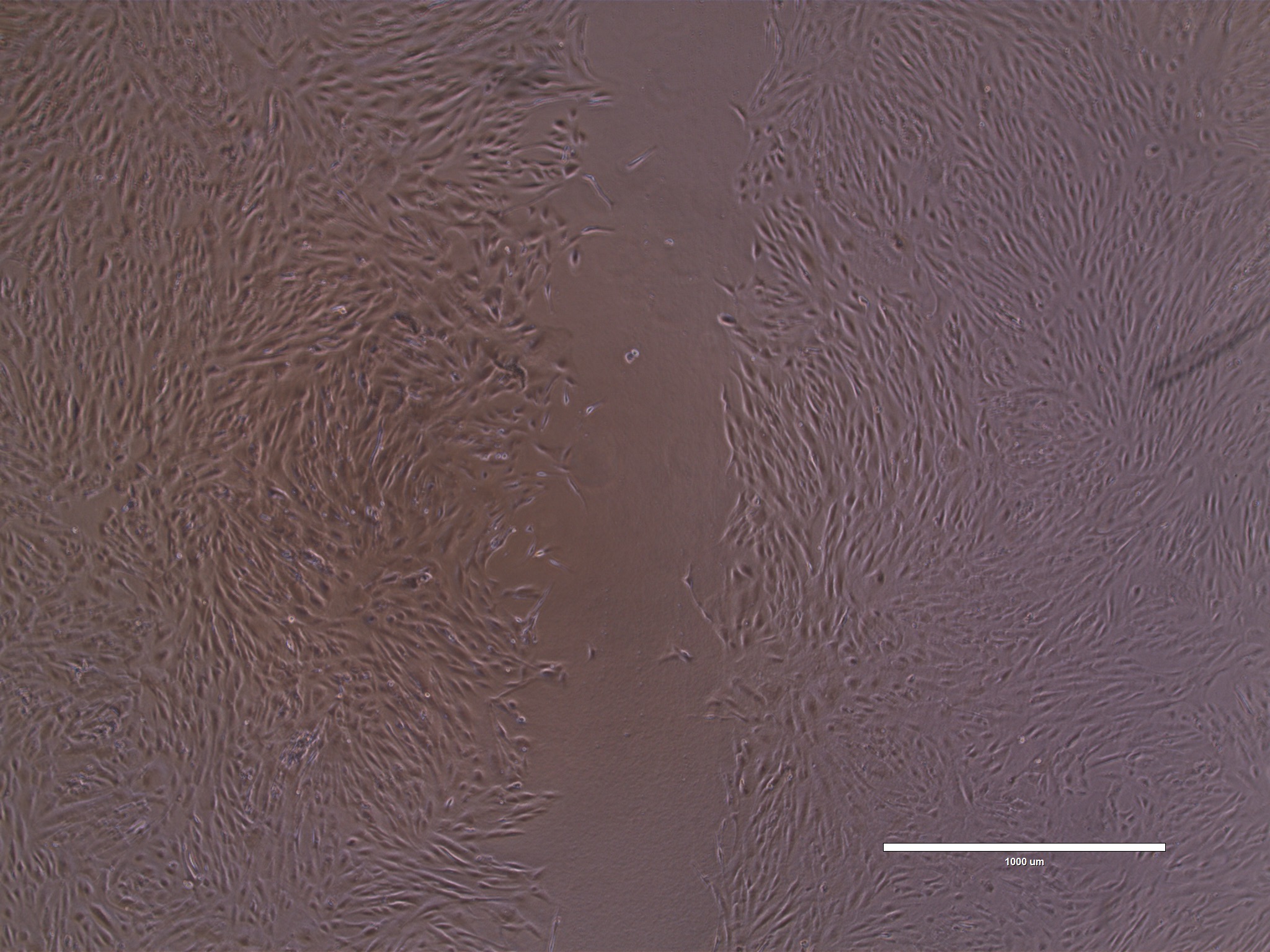


**Original Fig 5 (PB3-bdx-BB)**


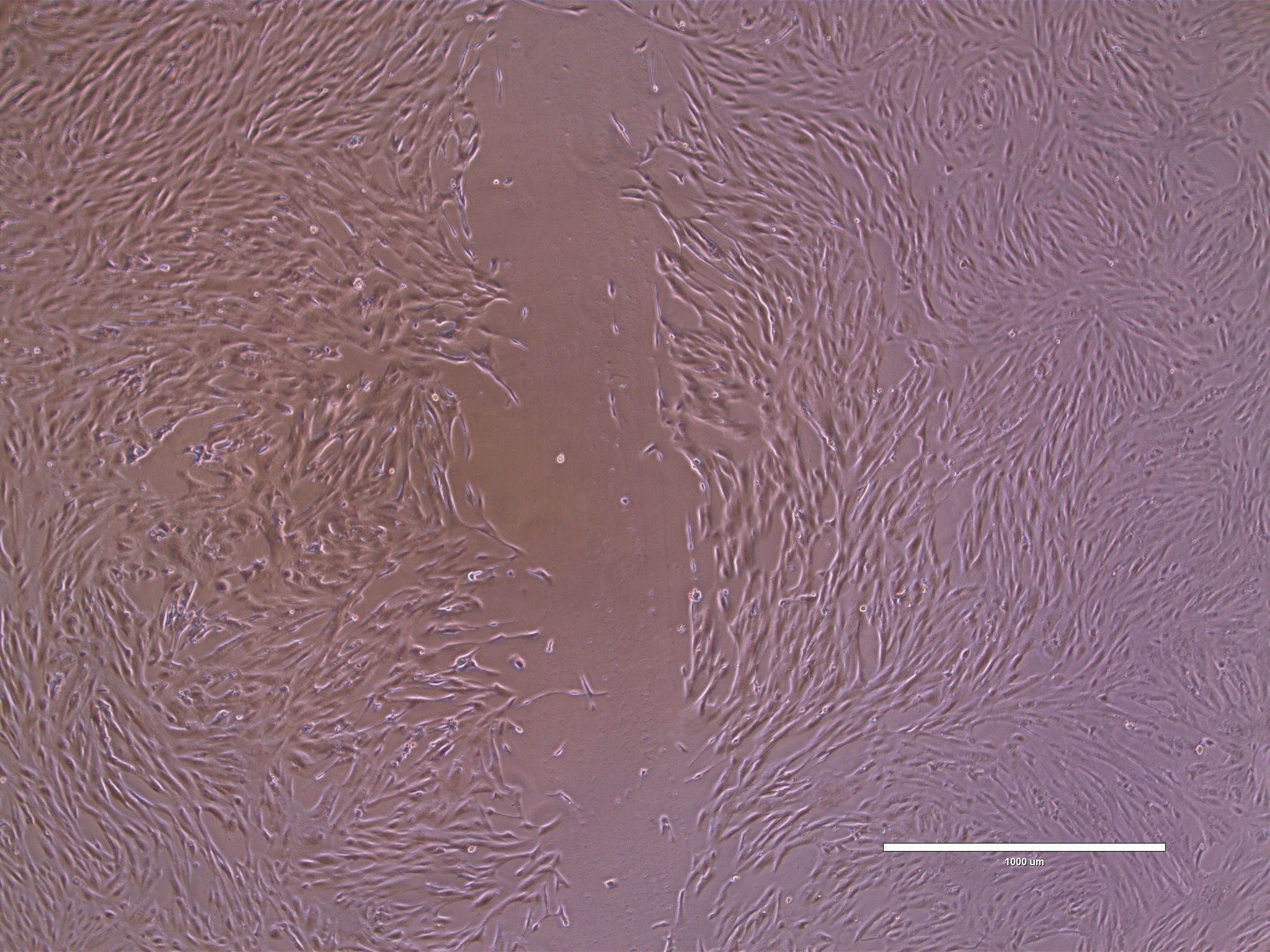


**Original Fig. 6**


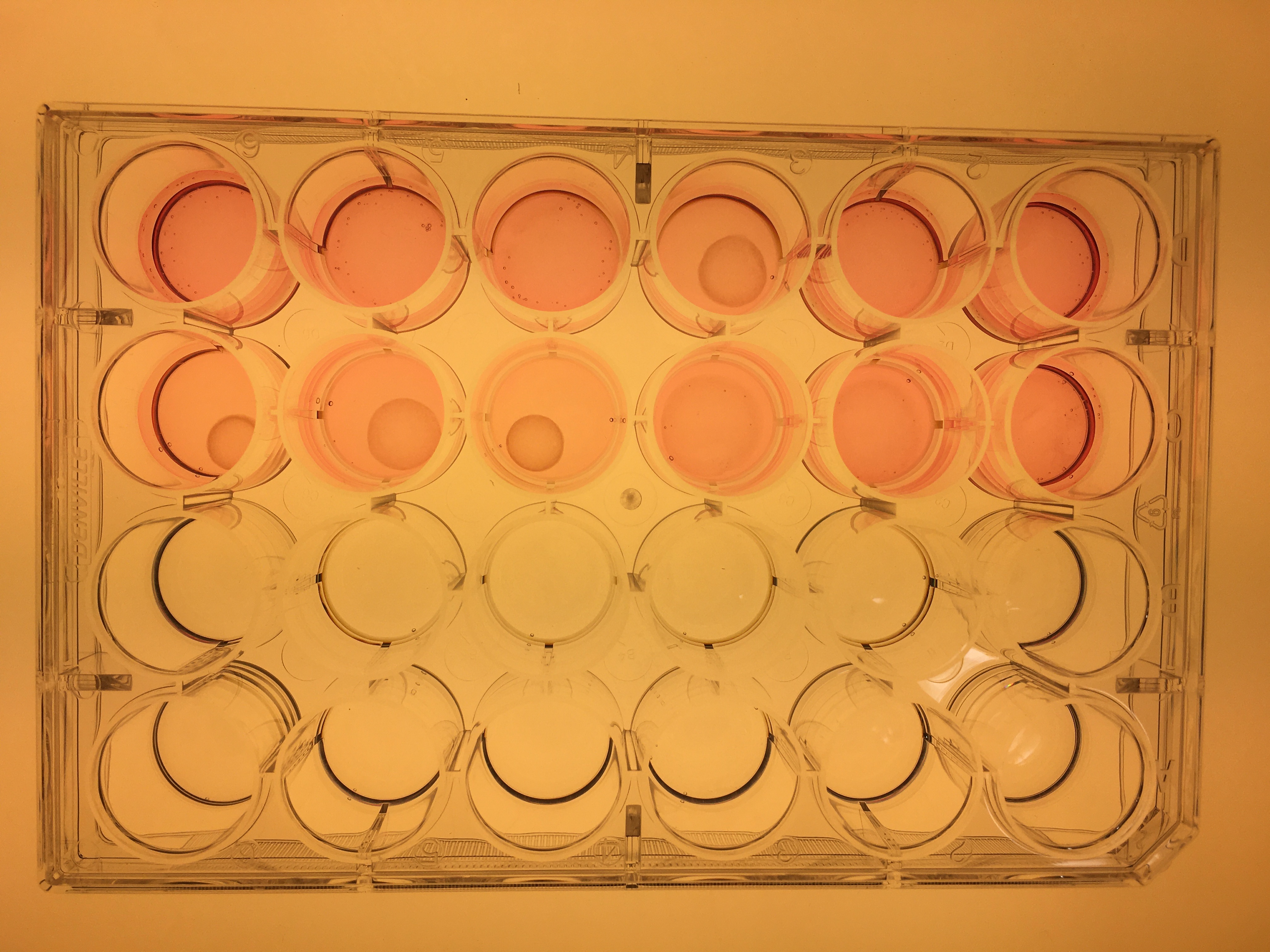


Original initial figure for the migration assay


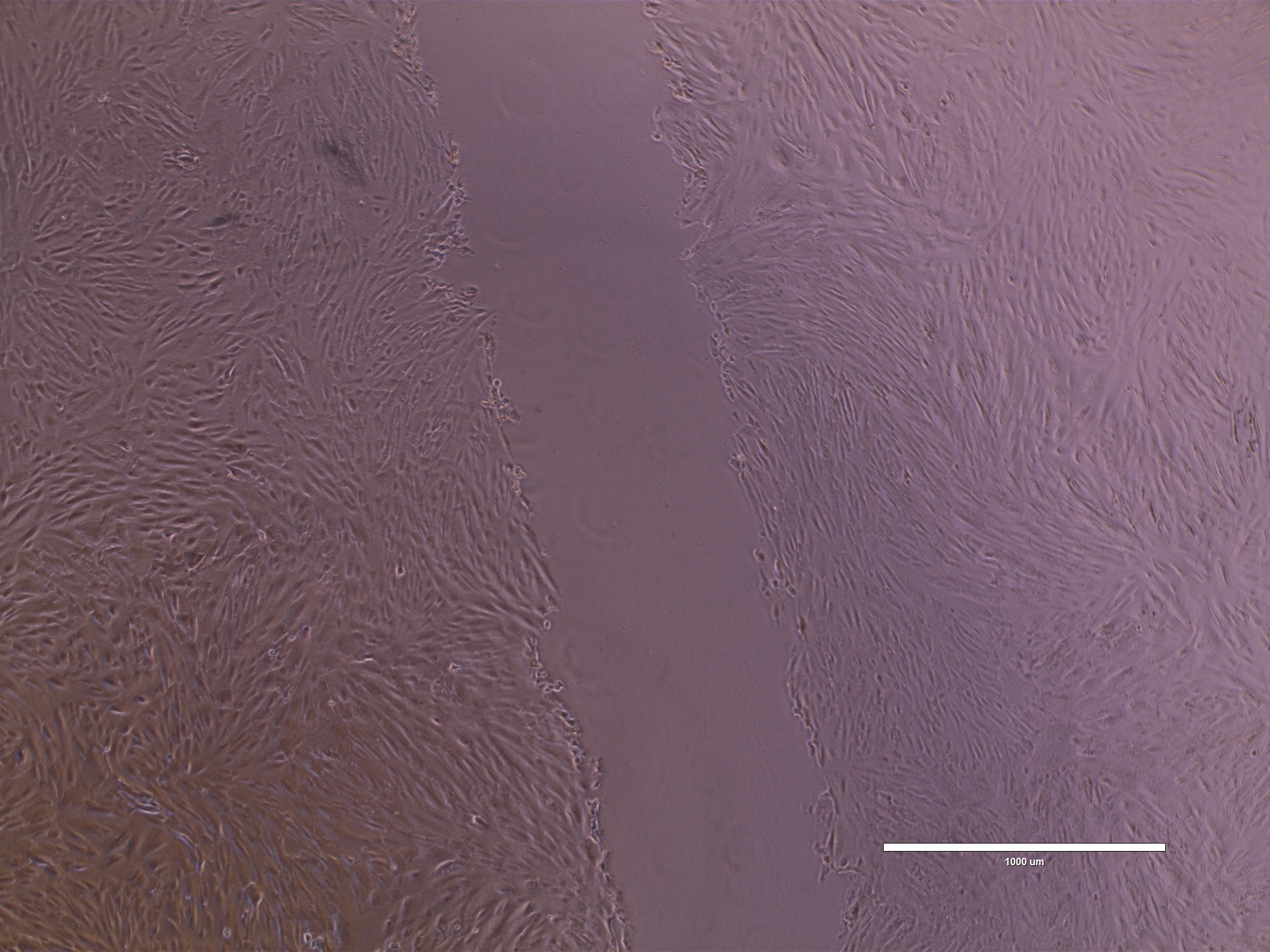

Supplement: Supplementary file 1 — Supplementary Material 1 [file 12886_2023_3089_MOESM1_ESM.docx]
