# Supplementary figures and images for: Ligand-independent activation of platelet-derived growth factor receptor β promotes vitreous-induced contraction of retinal pigment epithelial cells
Source: BMC Ophthalmol. 2023 Aug 3;23:344. doi: 10.1186/s12886-023-03089-8 (PMC10401781; doi:10.1186/s12886-023-03089-8)

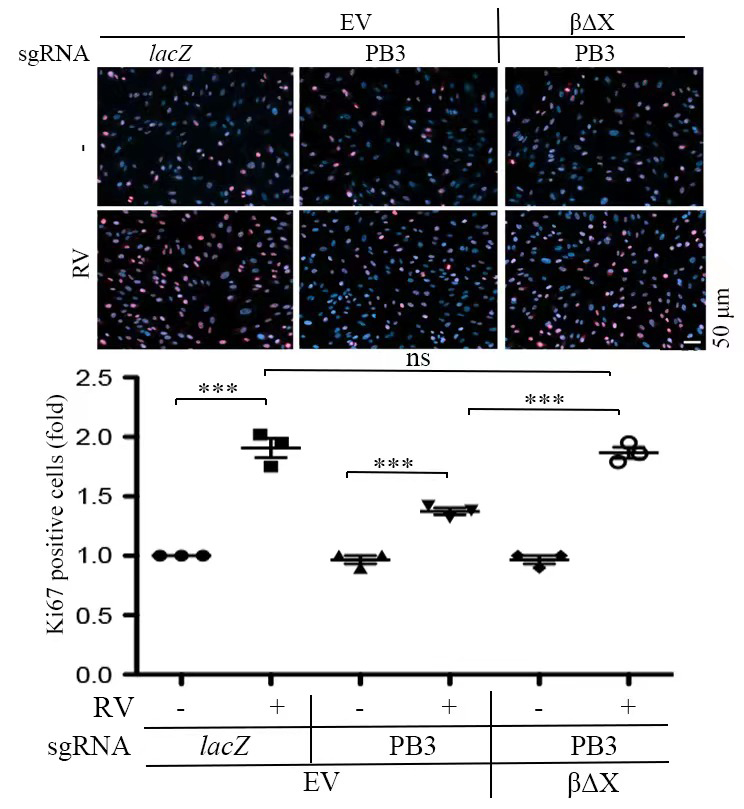

Supplement: Supplementary file 2 — Supplementary Material 2 [file 12886_2023_3089_MOESM2_ESM.jpg]
